# MTFMT deficiency correlates with reduced mitochondrial integrity and enhanced host susceptibility to intracellular infection

**DOI:** 10.1038/s41598-020-68053-8

**Published:** 2020-07-07

**Authors:** Jung-Hwa Seo, Cheol-Sang Hwang, Joo-Yeon Yoo

**Affiliations:** Department of Life Sciences, Pohang University of Science and Technology (POSTECH), Pohang, 790-784 Republic of Korea

**Keywords:** Mitochondria, Innate immunity

## Abstract

Mitochondria behave as functional and structural hubs for innate defense against intracellular infection. While the mitochondrial membrane serves as a platform for the assembly of signaling complexes activated by intracellular infection, various danger molecules derived from impaired mitochondria activate innate signaling pathways. Using methionyl-tRNA formyl transferase (MTFMT)-deficient cells, which exhibit impaired mitochondrial activity, we examined the role of mitochondrial integrity in regulating innate defense against infection. Since MTFMT functions at the early steps of mitochondrial translation, its loss was expected to cause defects in mitochondrial activity. Under transient MTFMT gene silencing conditions, we observed shortened mitochondria along with reduced activity. MTFMT-silenced cells were more susceptible to intracellular infection, as examined by infection with RNA viruses and the intracellular bacterium *Shigella flexneri*. In support of this observation, MTFMT-silenced cells possessed lowered basal NF-κB activity, which remained low after *S. flexneri* infection. In addition, the mitochondrial accumulation of evolutionarily conserved signaling intermediate in Toll pathway (ECSIT), an adaptor protein for NF-κB activation, was significantly decreased in MTFMT-silenced cells, explaining the reduced NF-κB activity observed in these cells. Since impaired mitochondria likely release mitochondrial molecules, we evaluated the contribution of mitochondrial *N*-formyl peptides to the regulation of bacterial infection. Transient transfection of mitochondrial-derived *N*-formyl peptides favored *S. flexneri* infection, which was accompanied by enhanced bacterial survival, but did not affect host cell viability. However, transient transfection of mitochondrial-derived *N*-formyl peptides did not affect basal NF-κB activity. Altogether, these data suggest that the integrity of mitochondria is essential to their proper function in protecting against infection, as intact mitochondria not only block the release of danger molecules but also serve as signaling hubs for the downstream NF-κB pathway.

## Introduction

As the mitochondrion, one of the largest subcellular organelles, orchestrates a variety of cellular processes, congenital or acquired mitochondrial defects participate in the development of several diseases^1^. When examining parts of the eukaryotic cell, the extent to which the cellular immune response is activated often correlates with mitochondrial activity^2^. Such activity can be determined by the following properties: mitochondrial morphology, which is controlled by fusion and fission processes; mitochondrial number, which is regulated through mitochondrial biogenesis and degradation; and mitochondrial compositional changes, which are caused by the leakage of mitochondrial molecules^3–5^. Changes in these properties at the molecular level alter the susceptibility of cells under pathophysiological conditions; therefore, understanding mitochondrial behavior is crucial to studying the development of various diseases at the organism level.

How the regulation of immune responses is associated with mitochondrial activity has been widely studied in different ways. Furthermore, the correlation of immune response regulation with mitochondrial structural integrity is an attractive subject due to the formation of an immune signalosome on the mitochondrial membrane. Mitochondrial antiviral-signaling protein (MAVS), a representative adaptor molecule on the mitochondrial membrane, serves as a signaling hub that links the activated cytosolic receptor retinoic acid-inducible gene I (RIG-I)/melanoma differentiation-associated protein 5 (MDA-5) with the downstream signaling complex for IRF and NF-κB activation^6–9^. Similarly, ECSIT, another type of cytosolic adaptor protein functioning in mitochondria, binds TNF receptor-associated factor 6 (TRAF6), which mediates IKK activation and NF-κB target gene expression^10^. On the mitochondrial membrane, ECSIT is also known to physically interact with MAVS, which leads to the activation of NF-κB- and IRF3/7-dependent gene expression^11^.

Occasionally, disruption of the mitochondria induces the release of mitochondrial damage-associated molecular patterns (DAMPs), which can lead to the activation of innate immune responses^5^. Mitochondrial DNA induces an inflammatory response by binding not only TLR9, a membrane surface receptor^12^, but also cytosolic NOD-like receptor pyrin domain-containing protein 3 (NLRP3) with the aid of mitochondrial reactive oxygen species (ROS)^13^. Similarly, *N*-formyl peptides synthesized in the mitochondria alert the immune system by binding formyl peptide receptor (FPR)^14,15^. However, whether these mitochondrial molecules inhibit pathogens or the host remains controversial.

Because mitochondria account for a considerable proportion of the volume of eukaryotic cells, it is not surprising that the regulation of cellular processes is highly dependent on the mitochondrial status. Based on the importance of mitochondrial structural integrity, we questioned the extent to which mitochondrial defects affect cellular susceptibility to pathogenic infection. In this study, we introduced a mitochondrial defect by altering the gene expression of methionyl-tRNA formyltransferase (MTFMT). MTFMT is a key mitochondrial protein during the mitochondrial translational process that adds a formyl group onto methionyl-tRNA to produce the mitochondrial-specific translational initiator tRNA^fMet^^16^. Inappropriate MTFMT activity not only causes imbalance in the mitochondrial proteome but also influences the maintenance of mitochondria. Furthermore, mutations in the MTFMT gene have been associated with progressive neurodegenerative disorders, such as Leigh syndrome^17,18^. At the cellular level, fibroblasts obtained from Leigh syndrome patients exhibit decreased levels of tRNA^fMet^ and impaired mitochondrial translational efficiency, which eventually leads to abnormal assembly of the mitochondrial OXPHOS complex^17^. Interestingly, overexpression of MTFMT had a dominant negative effect on the assembly of this complex, suggesting that MTFMT-mediated regulation of the translational initiation step is critical for the functional activity of mitochondria^19^.

In this study, using MTFMT-deficient cells with defective mitochondria under basal conditions, the significance of mitochondrial integrity in regulating the innate defense against infection was elucidated.

## Results

### MTFMT deficiency induces mitochondrial impairments

**Figure 1:**
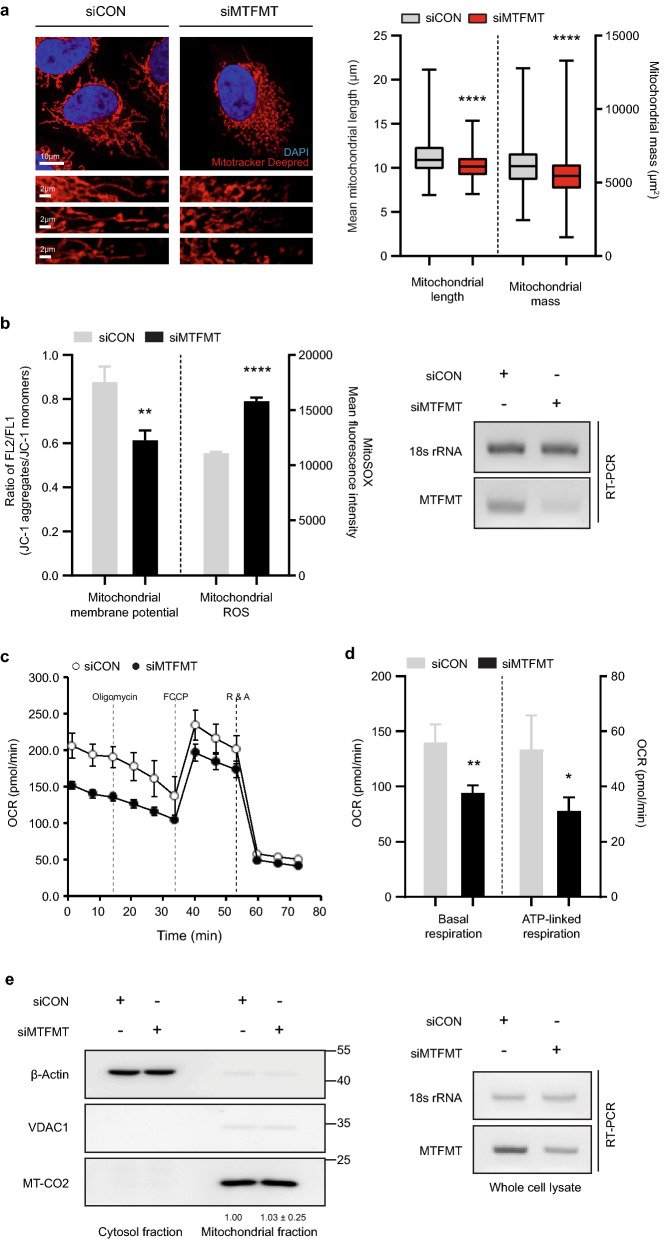
MTFMT deficiency disrupts the integrity of mitochondria. **(a)** HeLa cells were transfected with control or MTFMT siRNA and stained with MitoTracker Deep Red (400 nM) and DAPI. Four representative images of each group are shown (left). To statistically analyze mitochondrial length and mass, mitochondrial network analysis (MiNA), a macro for the measurement of mitochondrial morphology, was used to analyze > 400 cells collected from three independent experiments (right). The error bars indicate SDs (n = 459 for siCON and n = 424 for siMTFMT), and significance was assessed by an unpaired t-test. ****, P < 0.0001. **(b)** HeLa cells were transfected with control or MTFMT siRNA and stained with JC-1 or MitoSOX to measure mitochondrial membrane potential and mitochondrial ROS, respectively. The fluorescence intensity of JC-1 aggregates, JC-1 monomers and MitoSOX was measured by FACS (left). The error bars indicate SDs, and significance was assessed by an unpaired t-test. **, P < 0.01; ****, P < 0.0001. The MTFMT knockdown efficiency was assessed by RT-PCR, and representative data are shown after the inversion of an agarose gel image (right). For the original gel image, see Supplementary Fig. S4. **(c,d)** The cellular OCR in the control or MTFMT siRNA-transfected HeLa cells was measured using a Seahorse XFe96 analyzer. The parameters of basal respiration (**d**, left) and ATP-linked respiration (**d**, right) were automatically calculated by the Seahorse XF Mito Stress Test Report Generator based on the data in **(c)**. The error bars indicate SDs (n = 3 for siCON and n = 4 for siMTFMT), and significance was assessed by an unpaired t-test. *, P < 0.05; **, P < 0.01. **(e)** HeLa cells were transfected with control or MTFMT siRNA and fractionated to obtain mitochondrial and cytosolic fractions. To analyze mitochondrial translation efficiency, the levels of mitochondrial proteins synthesized in either mitochondria or the cytoplasm were measured by an immunoblotting assay (left). For the full blot image, see Supplementary Fig. S5a. The MTFMT knockdown efficiency was assessed by RT-PCR, and representative data are shown after the inversion of an agarose gel image (right). For the original gel image, see Supplementary Fig. S5b.

Mitochondria continuously undergo fusion and fission processes to properly respond to different cellular environments^4^. When cells become stressed, which induces mitochondrial damage, the mitochondrial quality control system is turned on, immediately activating the fission process to segregate unhealthy mitochondria from groups of healthy mitochondria (Supplementary Fig. S1). To examine whether a transient deficiency of MTFMT induces mitochondrial damage, we measured mitochondrial morphological parameters, such as length and mass, using a macro for mitochondrial network analysis (MiNA) to evaluate confocal microscopy images of mitochondria through an ImageJ plug-in^20^. Compared to control small interfering RNA (siRNA)-transfected cells (N = 459; mitochondrial length M = 11.34; SD = 2.24), MTFMT-deficient cells (N = 424; M = 10.20; SD = 1.46) possessed significantly shorter mitochondria (Fig. 1a). Similarly, the mitochondrial mass was reduced in MTFMT-deficient cells (N = 424; mitochondrial mass M = 5,470; SD = 1525.04) compared to that observed in control cells (N = 459; M = 6,194; SD = 1,475.22) (Fig. 1a). Knockdown levels of MTFMT protein were separately confirmed using an immunoblotting assay (Supplementary Fig. S2). We also measured the functionality of mitochondria based on their membrane potential, which can be directly assessed by determining the ratio of an aggregated form of JC-1 dye trapped in healthy mitochondria relative to the monomeric form of JC-1 outside mitochondria. The ratio value was reduced by 30% when MTFMT expression was silenced (Fig. 1b), indicating that the integrity of the mitochondria was impaired when normal levels of MTFMT expression were not maintained. FCCP is a potent mitochondrial uncoupler that dissipates mitochondrial membrane potential by augmenting proton permeability across the mitochondrial inner membrane. Although the addition of FCCP reduced the mitochondrial membrane potential, MTFMT silencing did not lead to a further decrease in mitochondrial membrane potential (Supplementary Fig. S3a). In addition, levels of mitochondrial ROS, products of an abnormal operation of the mitochondrial electron transport chain (ETC) system, were similarly elevated in MTFMT-silenced cells (Fig. 1b). As a control, we observed the levels of mitochondrial ROS increased by the addition of FCCP in dose-dependent manner (Supplementary Fig. S3b).

Next, the functionality of mitochondria was assessed by measuring the cellular oxygen consumption rate (OCR). Following the sequential addition of oligomycin, FCCP, and rotenone/antimycin A, which target mitochondrial electron transport chains, the OCR values of control- or MTFMT-silenced cells were measured using Seahorse XFe Analyzers. In every step with added chemical, MTFMT-silenced cells behaved similarly to control cells, but with relatively lower OCR value (Fig. 1c). The results showed that in MTFMT-silenced cells, the basal and the maximal respiratory capacities, representing OCR values taken before oligomycin treatment and after FCCP treatment, respectively, were significantly decreased (Fig. 1d). In addition, the ATP production capacity of MTFMT-silenced cells, measured by ATP-linked respiration (OCR_Oligomycin_ − OCR_FCCP_), was also significantly reduced (Fig. 1d). The cellular concentrations of ATP were also separately measured to support the above results (Supplementary Fig. S3c and d). These results suggest both structural and functional alterations of mitochondria occurred in MTFMT-silenced cells. Since MTFMT functions during mitochondrial translation processes, we suspected that the general mitochondrial translation efficiency may have been altered. To test this hypothesis, the steady-state levels of mitochondrial proteins were examined (Fig. 1e). We selected voltage-dependent anion-selective channel 1 (VDAC1) as a chromosomal DNA-encoded mitochondrial protein and MT-CO2 as a mitochondrial DNA-encoded mitochondrial protein. However, the levels of neither protein differed between the control- and MTFMT-silenced cells, indicating that although MTFMT functions during mitochondrial translation, its transient deficiency does not markedly affect translational efficiency.

### MTFMT deficiency weakens the cellular response against pathogenic infections

**Figure 2:**
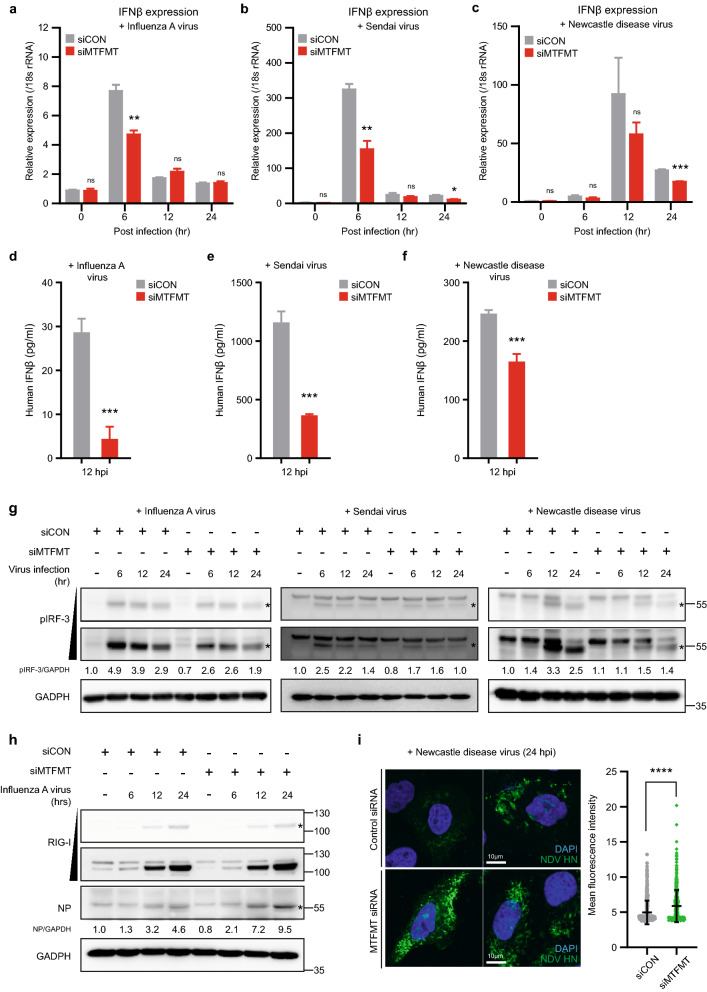
MTFMT deficiency affects cellular susceptibility to RNA virus infections. **(a)** HeLa cells transfected with control or MTFMT siRNA were infected with influenza A virus (PR8) and harvested at the indicated time points for real-time PCR analysis of IFNβ. **(b)** HeLa cells transfected with control or MTFMT siRNA were infected with Sendai virus and harvested at the indicated time points for real-time PCR analysis of IFNβ. **(c)** HeLa cells transfected with control or MTFMT siRNA were infected with Newcastle disease virus and harvested at the indicated time points for real-time PCR analysis of IFNβ. **(d–f)** Forty-eight hours after siRNA transfection, HeLa cells were infected with influenza A virus **(d)**, Sendai virus **(e)**, or Newcastle disease virus **(f)** for 12 h. Cell medium was harvested for quantifying the human IFNβ concentration as described in the Materials and Methods. The error bars of graphs from a-f indicate SDs, and significances were assessed by an unpaired t-test. *, P < 0.05; **, P < 0.01; ***, P < 0.001. **(g)** HeLa cells transfected with control or MTFMT siRNA were infected with influenza A virus (left), Sendai virus (middle) or Newcastle disease virus (right) and harvested for immunoblotting assays at the indicated time points. To detect phosphorylated IRF3, membranes were incubated with Tris-buffered saline (TBS) containing 0.05% Tween-20 during immunoblotting. For the full blot image, see Supplementary Fig. S6a-c. **(h)** HeLa cells transfected with control or MTFMT siRNA were infected with influenza A virus (PR8) and harvested for immunoblotting assays at the indicated time points. The membranes were incubated with phosphate-buffered saline (PBS) containing 0.05% Tween-20 during immunoblotting. For the full blot image, see Supplementary Fig. S6d. **(i)** HeLa cells transfected with control or MTFMT siRNA were infected with Newcastle disease virus. Twenty-four hours after virus infection, HeLa cells were stained with anti-viral HN protein antibody followed by anti-mouse Alexa 488. Nuclei were stained with DAPI (left). To quantify the degree of viral replication, the fluorescence intensity of the HN protein (> 500 cells) was measured using ImageJ (right). The error bars indicate SDs (siCON, N = 554; fluorescence intensity M = 4.965; SD = 1.69; siMTFMT, N = 562; M = 5.877; SD = 2.28), and significance was assessed by an unpaired t-test. ****, P < 0.0001.

As a primary target organelle and central hub for innate immunity, mitochondria are known to mediate cellular host responses upon both virus and bacterial infection^21^. Since we observed altered mitochondrial structure and function in MTFMT-deficient cells, we wondered whether the cellular response to intracellular infection was similarly altered. To this end, we infected HeLa cells with RNA viruses, including influenza A virus, Sendai virus, or Newcastle disease virus, the host response to which depends on cytosolic RIG-I-MAVS signaling pathways in mitochondria. MTFMT-silenced cells exhibited a weakened cellular response against viral infections, as judged by lower RNA expression levels of type I IFN (Fig. 2a–c) and decreased protein secretion (Fig. 2d–f) compared to that observed in control cells. IRF-3, a key regulator of type I IFN gene expression, was also less activated upon viral infections in MTFMT-silenced cells (Fig. 2g). In agreement with the cellular response against infection, the amounts of intracellular virus, measured by viral proteins, were significantly higher in MTFMT-silenced cells at 24 h post infections (Fig. 2h and i). However, in contrast to IFNβ, the production of IL-6 upon viral infection was not significantly different between the control- and MTFMT-silenced cells (Supplementary Fig. S7).

**Figure 3:**
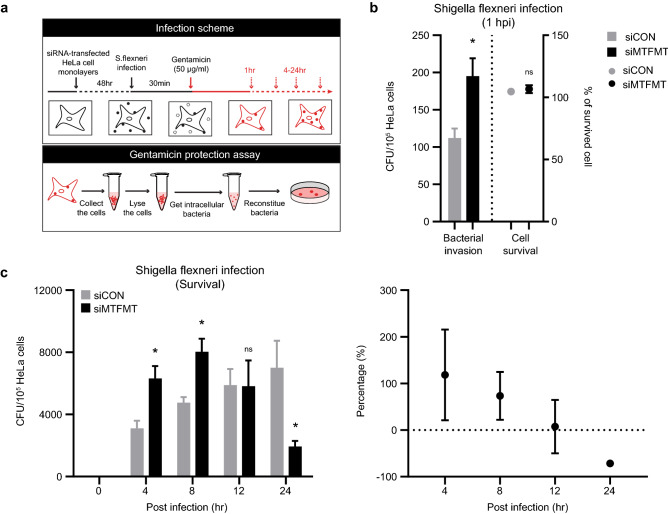
MTFMT deficiency affects cellular susceptibility to *S. flexneri* infection. **(a)** Illustration of the experimental scheme. HeLa cells were infected with *Shigella flexneri* (MOI = 10) for 30 min and then incubated in gentamicin-containing medium for the indicated lengths of time. Cell lysates were then harvested after 1 h or 4–24 h after infection and tested for intracellular bacterial invasion or survival, respectively, using a gentamicin protection assay. A detailed protocol is described in the “Materials and method” section. **(b)** One hour after *Shigella flexneri* infection, the number of live cells were counted using a hemocytometer, and then the HeLa cells were lysed with a 0.1% Triton X-100 solution and examined to measure bacterial invasion. The number of colony-forming units (CFUs) of *Shigella flexneri* obtained from cell lysates is shown (left). Circles mark the percentages of live cells under infection conditions (relative to mock infected conditions; right). The error bars indicate SEMs from three independent experiments, and significance was assessed by an unpaired t-test. *, P < 0.05. **(c)** Four to 24 h after *Shigella flexneri* infection, HeLa cells were lysed and examined to measure intracellular bacterial survival. The graph on the left indicates *Shigella flexneri* CFUs*,* and the graph on the right indicates the relative change in CFUs between control and MTFMT siRNA-transfected cells. Right and left panel use same data set. The error bars indicate SEMs (n = 3), and significance was assessed by an unpaired t-test. *, P < 0.05.

In addition to cellular responses against intracellular viral infection, increasing evidence also suggests that mitochondria play crucial roles during the cellular response against bacterial infection. Therefore, we also tested the effect of MTFMT deficiency during bacterial infection, using a *Shigella flexneri* infection system. *S. flexneri* is known to infect and replicate within the cytoplasm of epithelial cells^22^, and the number of replicating intracellular *S. flexneri* can be quantified using a gentamicin protection assay (Fig. 3a). *S. flexneri*-infected host cells were chased with gentamicin-containing media for the indicated times, and cell lysates were then plated on agar plates containing Congo red. The obtained colony numbers indicate the number of bacteria in proliferative and healthy states within the infected cells. To assess the initial bacterial invasion efficiency, bacteria-infected HeLa cells were chased with gentamicin-containing media for 1 h. An approximately two-fold increase in the number of intracellular bacteria during the very early stage of infection was detected in MTFMT-silenced cells compared to that observed in the control cells (Fig. 3b, left). Since cellular toxicity was not significantly altered by MTFMT silencing during the first hour of infection (Fig. 3b, right), we concluded that MTFMT deficiency increases the susceptibility of cells to *S. flexneri* infection.

Next, we measured the kinetics of intracellular *S. flexneri* proliferation under normal and MTFMT-deficient conditions. To this end, *S. flexneri-*infected cells were cultured in gentamicin-containing medium for an additional 4 to 24 h, and proliferating intracellular bacteria were counted using the gentamicin protection assay. In the control siRNA-transfected cells, the number of intracellular *S. flexneri* increased consistently over time, and proliferating intracellular bacteria were detected even after 24 h of infection (Fig. 3c, left). In contrast, MTFMT-silenced cells showed a different kinetics pattern of intracellular *S. flexneri* proliferation. During the early stage of infection, more proliferating bacteria were detected in MTFMT-silenced cells than in the control cells. However, the number of proliferating intracellular *S. flexneri* plateaued approximately 8 h after infection and then decreased (Fig. 3c, left). Consequently, substantially fewer intracellular *S. flexneri* were detected in MTFMT-silenced cells than in control cells at the later stage of infection (Fig. 3c, right). To determine whether MTFMT deficiency along with mitochondrial alterations leads to massive cell death at the later stage of infection, which may explain the peculiar kinetics of intracellular bacteria within, we next examined the occurrence of cell death. Under the non-infection conditions, programmed cell death accompanied by cytochrome C release was not observed in either the control or MTFMT-deficient cells (Supplementary Fig. S8a). Even throughout the entire period of infection, live cell numbers were not significantly different between control- and MTFMT-silenced cells (Supplementary Fig. S8b). These results indicate that MTFMT deficiency has a dual function in which pathogens and the host are favored during the early and later stages of infection, respectively.

### MTFMT deficiency results in decreased basal NF-κB activity

**Figure 4:**
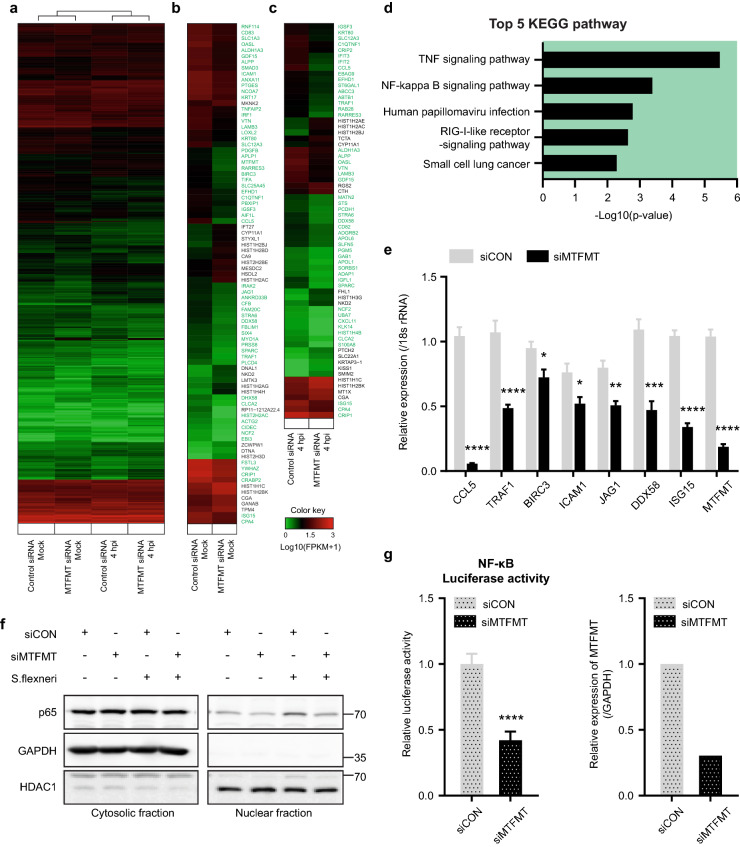
MTFMT deficiency reduces basal NF-κB activity. **(a)** Heatmap analysis of all genes in the control or MTFMT siRNA-transfected HeLa cells with or without *Shigella flexneri* infection. Log10 (FPKM + 1) ranges from 0 to 3. **(b, c)** Heatmap analysis of selected DEGs between the control and MTFMT siRNA-transfected HeLa cells without infection **(b)** and 4 h after infection **(c)**. A group of DEGs which expressions were downregulated in MTFMT-silenced cells was marked with green. **(d)** KEGG pathway analysis of downregulated genes in MTFMT-deficient cells compared to the control cells. **(e)** The mRNA expression levels of representative DEGs in HeLa cells transfected with control or MTFMT siRNA were separately tested by real-time PCR analysis. The error bars indicate SDs (n = 6), and significance was assessed by an unpaired t-test. *, P < 0.05; **, P < 0.01; ***, P < 0.001; ****, P < 0.0001. **(f)** Nuclear and cytosolic fractions of HeLa cells transfected with control or MTFMT siRNA were blotted for p65 before and after *Shigella flexneri* infection (MOI = 100, 45 min). For the full blot image, see Supplementary Fig. S10. **(g)** HeLa cells were cotransfected with siRNA and an NF-κB luciferase reporter plasmid. Forty-eight hours after transfection, a dual-luciferase assay was performed (left). The MTFMT knockdown efficiency was assessed by RT-PCR (right). The error bar indicate SDs (n = 6) and significance was assessed by an unpaired t-test. ****, P < 0.0001.

To gain insight into how MTFMT deficiency attenuates cellular immune responses during the early phase of infection, we performed a genome-wide transcriptome profiling assay. HeLa cells transfected with control or MTFMT siRNA were subsequently infected with *S. flexneri* for 4 h, after which RNA sequencing was performed for both infected and non-infected cells (Fig. 4a). By pairwise comparison of control and MTFMT siRNA-transfected and uninfected cells, 85 genes were identified as differentially expressed genes (DEGs) (fold-change ≥ 2, *p*-value ≤ 0.05) (Fig. 4b). Among these genes, the expression of approximately 71.8% (61 genes) was downregulated in MTFMT-silenced cells, with a maximum reduction of tenfold observed compared to the expression measured in control cells (Fig. 4b). The expression of these genes was similarly reduced in bacteria-infected MTFMT-silenced cells (Fig. 4c). The most interesting finding regarding these downregulated genes was their biological relationships with TNF signaling, NF-κB signaling, papillomavirus infection, and RIG-I-like receptor signaling pathways according to KEGG pathway enrichment analysis of DEGs (Fig. 4d)^23^. The expression of groups of genes involved in TNF-mediated NF-κB signaling (CCL5, TRAF1, BIRC3, ICAM1 and JAG1) and the RIG-I-like receptor signaling pathway (DDX58 and ISG15) were separately validated by real-time quantitative PCR (Fig. 4e). Another subset of genes (IL-6 and TNFα) that were not included among the DEGs shown in Fig. 4a, despite being known as NF-κB-responsive genes, were also examined for their mRNA expression level (Supplementary Fig. S9).

Among the identified DEGs, the reduced expression of NF-κB target genes was of particular interest. Since the basal and infection-induced expression levels of those NF-κB target genes were low in MTFMT-silenced cells, and the NF-κB signaling pathway is a well-characterized central player that controls the host response against intracellular infection^24^, we hypothesized that MTFMT-silenced cells have intrinsically lower basal NF-κB activity. The low levels of basal NF-κB activity may explain the reduced viral and bacterial responses, especially during an early phase of infections, in MTFMT-silenced cells. To test this hypothesis, we assessed NF-κB activity by measuring the active portion of total p65, an active NF-κB component, in the nuclear fraction. Under basal conditions, slightly reduced amounts of p65 were observed in the nuclear fraction of MTFMT-silenced cells. In control cells, *S. flexneri* infection induced the translocation of p65 from the cytoplasm to the nucleus. In contrast, in MTFMT-deficient cells, the nuclear translocation of p65 was significantly inhibited until 90 min after infection (Fig. 4f and Supplementary Fig. S11). However, IκBα degradation was not clearly observed upon *S. flexneri* infection in our experimental setting (Supplementary Fig. S12). The activity of the NF-κB signaling pathway was separately tested using an NF-κB-responsive luciferase reporter construct, the activity of which was verified in control experiments (Supplementary Fig. S13). Using a luciferase reporter assay, reduced NF-κB activity in MTFMT-silenced cells was separately confirmed (Fig. 4g). These results suggest that MTFMT-silenced cells with defective mitochondria possess decreased NF-κB activity under basal conditions, which may explain their attenuated cellular responses against intracellular infection during the early stage of infection.

### Recruitment of the NF-κB-activating complex ECSIT to the mitochondrial membrane under basal conditions is reduced in MTFMT-deficient cells

**Figure 5:**
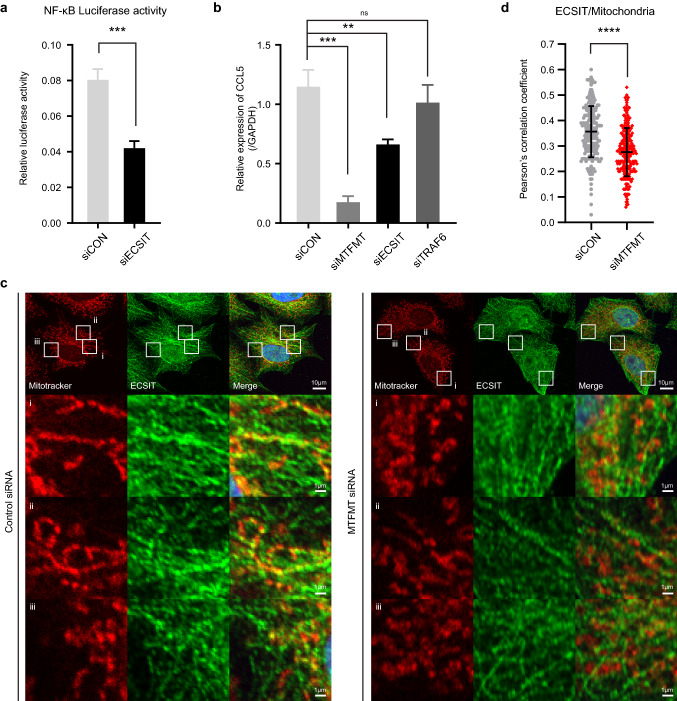
MTFMT deficiency impairs the recruitment of ECSIT onto mitochondria. **(a)** HeLa cells were cotransfected with siRNA and an NF-κB luciferase reporter plasmid. Forty-eight hours after transfection, a dual-luciferase assay was performed. **(b)** HeLa cells were transfected with the indicated siRNA and harvested for real-time PCR analysis. The error bars in the graphs from **(a)** and **(b)** indicate SDs, and significances were assessed by an unpaired t-test. **, P < 0.01; ***, P < 0.001. **(c)** HeLa cells were transfected with control (left) or MTFMT (right) siRNA and stained for mitochondria with MitoTracker Deep Red (red), endogenous ECSIT (green) and nuclei with DAPI (blue). (i–iii) Enlarged images of the indicated white boxes in the first row. **(d)** To statistically analyze ECSIT occupancy on mitochondria, Pearson’s correlation coefficient analysis of ECSIT on mitochondria was performed using > 200 cells from confocal microscopy images collected from several independent experiments. The error bar indicate SDs (n = 216 for siCON and n = 233 for siMTFMT), and significance was assessed by an unpaired t-test. ****, P < 0.0001.

To understand the molecular mechanisms associated with the reduced basal NF-κB activity in untreated MTFMT-silenced cells, we hypothesized that signaling complex formation on the mitochondrial membrane may be altered in cells lacking MTFMT expression. The detection of basal levels of NF-κB activity in control cells (Fig. 4f) suggests that spontaneous NF-κB signaling complex formation may occur in unstimulated cells. As a possible candidate molecule that activates NF-κB under basal conditions, we evaluated ECSIT, which interacts with TRAF6 on mitochondria^10^. When ECSIT expression was silenced in unstimulated cells, basal levels of NF-κB activity were reduced, confirming that ECSIT plays a role in the activation of basal NF-κB (Fig. 5a). In agreement with the observed basal NF-κB activity, the basal expression level of CCL5, a target gene of NF-κB, was similarly reduced in ECSIT-silenced cells (Fig. 5b), suggesting that ECSIT plays a role in the activation of basal NF-κB-mediated signaling pathways. Using confocal microscopy, we then observed the endogenous localization of ECSIT protein (Supplementary Fig. S14). When mitochondria were co-stained with MitoTracker, substantial amounts of ECSIT protein overlapped with areas harboring mitochondria in control cells (Fig. 5c). Although ECSIT has been reported to be recruited to mitochondria upon TLR activation in macrophages^10^, we observed that substantial amounts of ECSIT were already located at mitochondria under basal conditions (Supplementary Fig. S15). In contrast, those areas showing overlap between ECSIT and mitochondria decreased in MTFMT-silenced cells under basal conditions (Fig. 5c,d). Statistical analysis of the differences in ECSIT- and mitochondrial staining intensity demonstrated that the correlation score was significantly reduced in MTFMT-silenced cells. Based on these observations, we postulate that mitochondrial defects in MTFMT-deficient cells may prevent the stable recruitment of ECSIT to mitochondria, which inhibits the efficient activation of the NF-κB signaling pathway under basal conditions.

### Cytosolic *N*-formyl peptides promote infection independent of the NF-κB pathway

**Figure 6:**
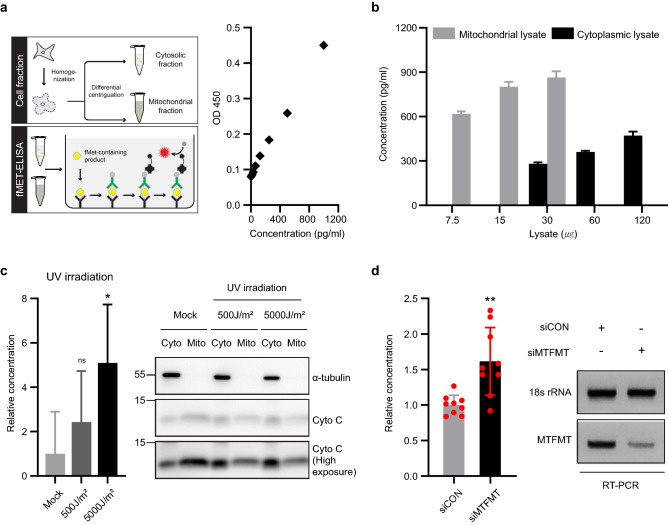
The cytosolic formyl peptide level increases in MTFMT-deficient cells. **(a)** Schematic diagrams of cell fractionation and the fMet ELISA assay (for a detailed protocol, see “Materials and methods”) and the standard curve from the fMet ELISA assay. **(b)** Formyl peptide levels in HeLa cell mitochondrial (7.5–30 μg) and cytosolic (30–120 μg) fractions were examined using an fMet ELISA. The error bars indicate the SD of triplicate experiments. **(c)** Formyl peptide levels in the cytosolic fraction (50 μg) of UV-irradiated HeLa cells assessed by fMet ELISA. The error bars indicate SD (n = 4), and significance was assessed using an unpaired t-test (left). *, P < 0.05. Cytochrome C and α-tubulin levels in mitochondrial (6 μg) and cytosolic (6 μg) fractions measured by an immunoblotting assay (right). For the full blot image, see Supplementary Fig. S17. **(d)** Formyl peptide levels in the cytosolic fraction (40–80 μg) of HeLa cells transfected with control or MTFMT siRNA were examined by an fMet CLIA assay (left). Representative RT-PCR data after inversion of the gel image showing the knockdown efficiency of the MTFMT gene (right). For the original gel image, see Supplementary Fig. S18. The error bars indicate SDs from three independent experiments, and significance was assessed by an unpaired t-test. **, P < 0.01.

In addition to reduced NF-κB/ECSIT formation on mitochondria, we explored additional effects caused by altered mitochondrial integrity. One hallmark of impaired mitochondria is a leakage of their contents, including mitochondrial DNA and peptides. Since we observed mitochondrial alterations in MTFMT-deficient cells, we wondered whether mitochondrial-derived DNA or peptides could be released from the impaired mitochondria. However, the amounts of detected cytosolic mtDNA were not significantly different (Supplementary Fig. S16). To measure mitochondrial peptides, we used a double-sandwich ELISA kit designed to detect *N*-formylmethionine (fMet) in serum. In this assay, a fMet-specific antibody, which is prebound to a reaction plate, can capture fMet-containing products (*N*-formyl peptide) in the sample up to 1,000 pg/ml (Fig. 6a). First, the mitochondrial and cytosolic fractions were separately harvested by differential centrifugation from HeLa cell lysates and then assayed for the presence of *N*-formyl peptides. In a test using a mitochondrial lysate (from 7.5 to 30 μg total), a positive correlation between the quantity of the lysates and fMet-reactive signal was observed (Fig. 6b). On average, 54.9 ± 23.3 pg of fMet-reactive signal per μg of mitochondrial lysate was detected. We next used four-fold excess amounts of cytosolic lysates (from 30 to 120 μg total) to detect any fMet-reactive signal. Although much less than that observed for mitochondrial lysates, significant levels of fMet-reactive signal (6.43 ± 2.4 pg/μg) were detected in the cytosolic fraction (Fig. 6b). Since this finding indicates that *N*-formyl peptides may be present in the cytoplasm, we next examined whether the fMet-reactive signal could be altered when mitochondria were disrupted by UV irradiation. Upon UV irradiation, where a clear cytosolic release of mitochondrial cytochrome C was observed, increased levels of fMet-reactive signal were detected (Fig. 6c). Therefore, using this assay, we measured fMet-reactive signal from the cytosolic fraction of MTFMT-silenced cells (Fig. 6d). From the repeated experiments, we clearly observed significantly higher levels of fMet-reactive signal in MTFMA-silenced cells compared to that observed in control HeLa cells. These results suggest that mitochondrial formyl peptide may be released from mitochondria in MTFMT-silenced cells, although the mechanism associated with this release remains unclear.

**Figure 7:**
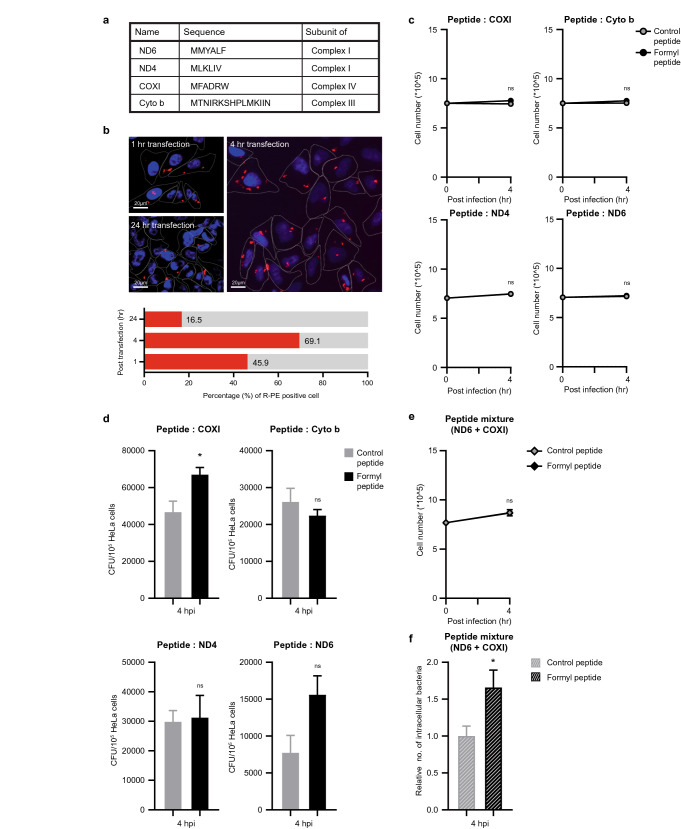
Transfection of synthetic *N*-formyl peptides affects cellular susceptibility to *Shigella flexneri* infection. **(a)** Details of the synthetic peptides used in this experiment. **(b)** Transfection efficiency of R-phycoerythrin (R-PE) (6 μg) into HeLa cells. **(c)** HeLa cells were transfected with the indicated synthetic peptides (control or formyl-containing peptides) and infected with *S. flexneri* (MOI = 10) for 4 h. The viability of peptide-transfected HeLa cells was determined by counting live cells at the indicated time point. **(d)** HeLa cells were transfected with the indicated synthetic peptides (control or formyl-containing peptides) and infected with *S. flexneri* (for COXI and Cyto b, MOI = 10; for ND4 and ND6, MOI = 20) for 4 h. The number of intracellular bacteria was determined by a gentamicin protection assay at the indicated time point. The error bars indicate SEMs (n = 3), and significance was assessed by an unpaired t-test. *, P < 0.05. **(e)** HeLa cells were transfected with a peptide mixture (ND6 + COXI) and infected with *S. flexneri* (MOI = 10) for 4 h. The viability of peptide-transfected HeLa cells was determined by counting live cells at the indicated time point. **(f)** HeLa cells were transfected with a peptide mixture (ND6 + COXI) and infected with *S. flexneri* (MOI = 10). The error bars indicate SEMs from three independent experiments, and significance was assessed by an unpaired t-test. *, P < 0.05.

Next, we assessed whether such mitochondrial-derived *N*-formyl peptide plays any roles during the host defense response against infection inside cells. To directly address this question, cells were transiently transfected with synthesized *N*-formyl peptides, and the effect of cytosolic *N*-formyl peptides on the defense response was assessed after *S. flexneri* infection. In this assay, through peptide transfection, we attempted to mimic MTFMT-silenced basal cell conditions. We used four different *N*-formyl peptides, where each sequence was derived from one of the mitochondrial proteins ND6, ND4, COXI, or Cyto b (Fig. 7a). Each control peptide had the same amino acid sequence, except for the absence of a formyl group on the first methionine residue. The transfection efficiency and stability of the peptides inside cells was examined using R-phycoerythrin (R-PE) transfection (Fig. 7b). The transfection of R-PE alone did not dramatically alter cell viability. However, the bacterial infection of control- or formyl peptide-transfected cells caused cell death at a later stage of infection (data not shown). Since peptide transfection along with bacterial infection was not observed to have a cytotoxic effect during the early stage of infection (Fig. 7c), we examined the regulatory effects of cytosolic *N*-formyl peptides during this early period in infection.

**Figure 8:**
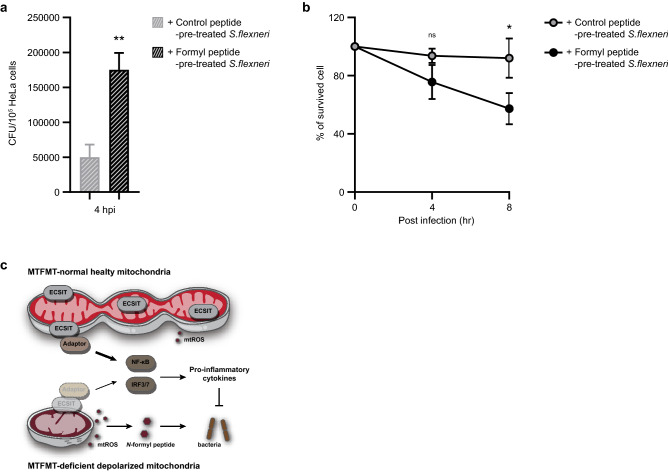
Mitochondrial *N*-formyl peptide enhances the replication of intracellular *Shigella flexneri.*** (a,b)** Bacterial pellets (MOI = 50) were incubated with a mixture of control or formyl peptides in PBS at 37 °C. After incubating for 30 min, bacterial suspensions were added to the cell medium to which HeLa cells had been seeded a day before. Intracellular bacteria and live cells were counted at the indicated time points. The error bars indicate SDs, and significance was assessed by an unpaired t-test. *, P < 0.05; **, P < 0.01. **(c)** Under normal-MTFMT healthy mitochondria conditions, mitochondria serve as a signaling complex platform that exhibits steady-state NF-κB activity. In addition, the leakage of danger molecule is minimized under these conditions. In MTFMT-deficient depolarized mitochondria, the assembly of the signaling complex on mitochondria is reduced, as exemplified by the mitochondrial adaptor protein ECSIT. In addition, danger molecule levels from impaired mitochondria likely increases, resulting in favorable conditions for intracellular infection.

When individual *N*-formyl peptides were transfected, their effects on intracellular bacterial survival were marginal, with the exception of the COXI-derived *N*-formyl peptide (Fig. 7d). However, the effect on bacterial survival was most significant when a mixture of COXI- and ND6-derived *N*-formyl peptides was used, following which an average bacterial survival rate of more than 60% was observed compared to that of control peptide-transfected cells (Fig. 7e and f). Finally, we examined whether cytosolic *N*-formyl peptides directly control basal NF-κB activity. However, basal NF-κB activity was not significantly altered by the transfection of either control or formyl peptides alone (Supplementary Fig. S19). Furthermore, when *S. flexneri* cells were pre-incubated with *N*-formyl peptides before infection, their invasive capacity and virulence were much stronger (Fig. 8a and b). These data indicate that mitochondrial-derived *N*-formyl peptides directly favor bacteria but not through regulating the host defense response mediated by the NF-κB signaling pathway.

## Discussion

In this study, we investigated the role of mitochondrial integrity in the defense against infection. Taking advantage of MTFMT-deficient cells that have both structurally and functionally impaired mitochondria but relatively intact mitochondria translation potential, we propose that mitochondrial integrity has dual functions: it is required for the proper assembly of signaling complexes, observed in this study by the NF-κB-activating ECSIT on mitochondria, and the blockage of mitochondrial DAMPs, observed in this study as mitochondrial *N*-formyl peptides, likely released from impaired mitochondria (Fig. 8c).

On the mitochondrial membrane, a signaling complex activates NF-κB or IRF assembly upon intracellular infection. One of the best-studied adaptor molecules on mitochondria is MAVS. RNA virus-infection triggers RIG-I activation through a conformational change, after which activated RIG-I moves into mitochondria to bind its adaptor protein MAVS. Since MAVS is a transmembrane protein, it forms a signaling complex on mitochondria and consequentially transmits the activating signal to downstream components. In contrast, ECSIT which functions as an adaptor protein in the Toll/IL-1 receptor-mediated signaling pathway, is primarily located in the cytoplasm^25^. However, upon ligand binding, TRAF6 ubiquitinates ECSIT for signal transmission, and their interaction can be detected on mitochondria^10^. The NF-κB pathway is known to function downstream of ECSIT. In this study, we observed that (1) MTFMT deficiency attenuates immunity-related signaling pathways, such as the NF-κB pathway; and (2) the mitochondrial accumulation of ECIST diminishes when mitochondrial structure and function is altered by MTFMT deficiency. Indeed, ECSIT has recently been shown to mediate a physical connection between RIG-I and MAVS on the mitochondria upon viral infection^11^. Since RIG-I-like receptor-signaling pathways are one of the affected KEGG pathways in our transcriptome analysis (Fig. 4d), we speculate that the interaction between RIG-I and MAVS could also be attenuated by MTFMT deficiency. In this case, MTFMT deficiency appears to inhibit upstream of MAVS, because “basal” expression and accumulation of MAVS on mitochondria was not significantly altered in MTFMT-deficient cells (Supplementary Fig. S20).

In this study, we tested the effects of cytosolic formyl peptides in response to infection. The mitochondrion is an intrinsically non-self organelle^26^ harboring many danger molecules that function during defense responses against infection by directly or indirectly stimulating innate immune responses^5,27^. Mitochondrial *N*-formyl peptides are structurally and functionally similar to bacterial formyl peptides. The binding of *N*-formyl peptides to FPRs in the plasma membrane leads to changes in calcium flux and MAPK pathway activation^28^. Consequent gene expression leads to the recruitment of immune cells around the site of infection or injury, which explains how *N*-formyl peptides act as chemoattractants^29,30^. The release of mitochondrial-derived molecules, which is typically accompanied by the breakdown of mitochondrial integrity, is a warning sign of danger^5,27^. The release of cytochrome c protein from mitochondria into the cytoplasm indicates a specific type of cell death, apoptosis. Furthermore, the release of mitochondrial peptides during the mitochondrial unfolded protein response (mtUPR) turns on the cellular stress response^31^. The mtUPR induces the accumulation of mitochondrial ROS and the cleavage and subsequent release of mitochondrial proteins. In *C. elegans*, the mitochondrial proteases LONP-1 and CLPP-1 participate in the cleavage process during the mtUPR^32,33^, and the resultant cleaved peptides are released into the cytoplasm through the peptide exporter HAF-1^34^. Consequently, ATFS-1, a major transcription factor, translocates to the nucleus, where it regulates the expression of stress-responsive genes^33^. The presence of mitochondrial-derived peptides has also been reported in the cytosol of *S. cerevisiae*^35^. In this study, we mimicked the cytosolic formyl peptide by transfection and assessed its role in the regulation of bacterial infection. Currently, the mechanism of this process remains unknown. However, we demonstrated that pre-existing mitochondrial *N*-formyl peptide favors bacterial survival.

In this study, we used four different types of synthetic peptides with methyl or formyl groups attached to their N-terminus. Each amino acid sequence was derived from mitochondrial proteins, which are translated inside of the mitochondrial matrix and not by the cytosolic translation machinery. Although 13 proteins can be synthesized in the mitochondria, only a subset of mitochondrial *N*-formyl peptides were effective. We chose ND6, ND4, and COXI because they were previously reported to show high agonistic activity against FPR1 and FPR2 based on their capacity to increase intracellular calcium flux^36^. We also tested the Cyto b peptide based on its ability to induce β-hexosaminidase release and chemotaxis in HL-60 cells^37^. Although all the peptides used in this study possessed an *N*-formyl methionine residue at their *N*-terminus, each peptide had a different effect (Fig. 5), suggesting that the formyl group itself is not sufficient to enhance bacterial virulence, although it is required. It will be of further interest to establish effective peptide parameters, such as peptide length, amino acid composition or order to tailor this effect.

In summary, mitochondrial integrity is crucial in mediating the defense response against infection. Healthy mitochondria exhibit dual functions by providing a physical platform for the stable assembly of signaling complexes and preventing the leakage of unwanted danger molecules from the mitochondria to the cytosol. The results of our study confirm that mitochondria are central organelles that regulate the immune response to infection and demonstrates the importance of maintaining mitochondrial health.

## Materials and methods

### Cell culture and transfection

HeLa cells were cultured in Dulbecco’s modified Eagle’s medium (DMEM; Lonza, cat. #12-604F, Walkersville, MD, USA) supplemented with 10% fetal bovine serum (FBS; Welgene, cat. #S001-01, Gyeongsan, Republic of Korea). For plasmid transfection, plasmid DNA (1 µg/35-mm dish) was mixed with Effectene reagent (Qiagen, cat. #301425, Valencia, CA, USA) and added to a dish in which cells had been seeded 12 h before. The cell medium was replaced with fresh complete medium after 6 h. siRNA oligonucleotides against human MTFMT (5′-CCACAAACAGUCACAAAUATT-3′ and 5′-UAUUUGUGACUGUUUGUGGTT-3′), human ECSIT (5′-AUUGAUGUCAAACUCGUAGTT-3′ and 5′-CUACGAGUUUGACAUCAAUTT-3′), human TRAF6 (5′-GCGCUGUGCAAACUAUAUATT-3′ and 5′-UAUAUAGUUUGCACAGCGCTT-3′) and control oligonucleotides were purchased from GenePharma (Shanghai, China). For siRNA transfection, siRNA (87.5 pmol/35-mm dish) was mixed with Lipofectamine 2000 reagent (Invitrogen, cat. #11668019, Carlsbad, CA, USA) and added to a dish containing cells. The cell medium was replaced with fresh DMEM containing 5% FBS after 10–12 h.

### Measurement of mitochondrial length and mass

For measurements mitochondrial morphological parameters, fluorescence images of mitochondria were obtained by confocal microscopy (FLUOVIEW FV3000; Olympus, Tokyo, Japan). Each image that contained only one cell was turned into a binary image (black/white) and subsequently analyzed using a ImageJ macro called MiNA. This plug-in automatically measures the volume and length of mitochondria based on white pixels.

### Mitochondrial stress assay

siRNA-transfected cells were trypsinized and recounted for reseeding in an XF96 cell culture microplate (Agilent, cat. #102416-100, Santa Clara, CA, USA) 36 h before the assay. XF base medium (Agilent, cat. #102353-100) was supplemented with 1 mM pyruvate, 2 mM glutamine, and 10 mM glucose, and the pH was adjusted to 7.4 immediately before the assay. Each mitochondrial inhibitor (Agilent, cat. #103015-100) was dissolved in assay medium (at the following concentrations: 100 µM oligomycin, 100 µM FCCP, and 50 µM rotenone/antimycin A) and loaded onto an XFe96 sensor cartridge (Agilent, cat. #102416-100). After incubating in a non-CO_2_ incubator for 1 h, the microplate and sensor cartridge were loaded onto a Seahorse XFe96 analyzer, and the assay was performed. Each parameter was automatically calculated by the Seahorse XF Mito Stress Test Report Generator.

### Cell fractionation

For nuclear/cytosolic fractionation, cells were lysed with sucrose buffer [10 mM Tris–HCl (pH 8.0), 320 mM sucrose, 3 mM CaCl_2_, 2 mM MgOAc, 0.1 mM EDTA, and 0.5% NP-40] and divided into cytosolic (supernatant) and nuclear (pellet) fractions by centrifugation at 2,400 rpm for 5 min at 4 °C. For mitochondrial/cytosolic fractionation, cells were lysed with mitochondrial isolation buffer [10 mM Tris–HCl (pH 7.5), 320 mM sucrose, and 1 mM EDTA], and the supernatant was obtained by centrifugation at 1,000 × *g* for 10 min at 4 °C. After one more round of centrifugation at 11,000 rpm for 15 min at 4 °C, the cytosolic (supernatant) and mitochondrial (pellet) fractions were obtained.

### Luminescent ATP detection assay

After the detachment of HeLa cells from tissue-culture plates by trypsinization, the cells were counted using an automated cell counter (CellDrop Cell Counters; DeNovix, Wilmington, DE, USA) to collect 4 × 10^5^ cells. A cell pellet was obtained by centrifugation, and the cells were subsequently resuspended in 1 ml of PBS. Then, the cells were collected by centrifugation and lysed with 1 × Passive lysis buffer (Promega, cat. #E1941, Madison, WI, USA) in a vertical microtube rotator for 15 min at 4 °C. The supernatant was separated from cell debris by centrifugation at 13,000 rpm for 5 min and then assayed to determine the cellular ATP concentration using a Luminescent ATP detection assay kit (Abcam, cat. # ab113849, Cambridge, UK).

### Bacterial strain and gentamicin protection assay

*Shigella flexneri* (2457 T) was a kind gift from Dr. Jae-Ouk Kim (International Vaccine Institute, Seoul, Korea). One day before infection, colonies were inoculated and incubated in 5 ml of tryptic soy broth (TSB) overnight. On the day of infection, the bacterial broth was diluted 1:100 in fresh TSB and incubated at 37 °C in a shaking incubator at 2,400 rpm until the OD value reached 0.6–0.8. After determining the bacterial dose, the bacterial pellet was obtained by centrifugation at 3,400 rpm for 10 min and then resuspended in 500 µl of PBS. The bacterial suspension was then added to a dish in which the cell medium had been replaced with fresh complete medium before infection. For the bacterial invasion assays, cells with the added bacterial suspension were centrifuged at room temperature at 1,800 rpm for 10 min and then incubated at 37 °C. After 30 min of incubation, the cells were washed three times with cold PBS and cultured in fresh complete medium containing 50 µg/ml gentamicin for 1 h. Cell pellets were then obtained by trypsinization, after which the pelleted cells were lysed, diluted in 0.1% Triton X-100 and spread onto TSB agar plates containing Congo red (Serva, cat. #27215.01, Heidelberg, Germany) to reconstitute intracellular bacteria. For the bacterial survival assay, infected cells were incubated in gentamicin-containing medium for 24 h.

### Viral infection

For viral infection assays, the cell medium was replaced with serum-free medium containing 10 TCID50/ml of influenza A virus (A/PR8/1934(H1N1)), 300 TCID50/ml of Sendai virus, or 15 TCID50/ml of Newcastle disease virus (NDV, La Sota). After 3 h of incubation at 37 °C, the medium was replaced with fresh DMEM containing 10% FBS, and cells were harvested for RNA extraction at various time points.

### Human IFNβ and IL-6 ELISA

HeLa cells were seeded at 2 × 10^5^ cells/well in six well tissue culture plate. Twelve hours after cell seeding, siRNA was transfected into cells with Lipofectamine 2000 reagent (Invitrogen, cat. #11668019). Forty-eight hours after siRNA transfection, the cell medium was replaced with serum-free medium containing each virus. After 3 h of incubation, the medium was replaced with fresh DMEM containing 10% FBS. The supernatants of virus-infected cells were collected at 12 h post infection and used to quantify the concentrations of human IFNβ and IL-6 using a Human IFNβ Quantikine ELISA kit (R&D Systems, cat. #DIFNB0, Minneapolis, MN, USA) and a Human IL-6 Quantikine ELISA kit (R&D Systems, cat. #D6050), respectively.

### Dual-luciferase reporter assay

HeLa cells were cotransfected with siRNA and luciferase reporter plasmids (pLuc-NF-κB with pRenilla-GAPDH) with Lipofectamine 3000 reagent (Invitrogen, #L3000015). The cell medium was replaced with fresh DMEM containing 10% FBS after 6 h, and a dual-luciferase reporter assay was performed according to the manufacturer’s instructions (Promega, #E1960).

### fMet ELISA and CLIA

For measurements of intracellular levels of formyl peptide, two ELISA kits were used: fMet ELISA (MyBioSource, cat. # MBS265039, San Diego, CA, USA) and fMet CLIA (Elabscience, cat. # E-CL-H0950, Houston, TX, USA). Cytosolic or mitochondrial fractions were quantified by Bradford assay. The measurement of fMet in the cellular lysates was conducted according to the manufacturer’s instructions. Briefly, appropriate amounts of sample were loaded into fMet-antibody-precoated 96-well plates and incubated for 90 min at 37 °C. After washing with wash buffer supplied by the manufacturer, biotinylated a fMet-antibody was added into the plate and incubated for 60 min at 37 °C. After washing, an avidin-conjugated solution was added and incubated for 30 min at 37 °C. After the final round of washing, color reagent was added, and the plate was incubated until a color change appeared (typically within 10 min). When the chromogenic reaction was completed, the OD value was read at 450 nm with an ELISA reader. For CLIA, after the final round of washing, luminol substrate was added, and relative light unit (RLU) values were determined using a luminescence microplate reader.

### Peptide transfection

Mitochondrial *N*-formyl peptides or control unformylated peptides were synthesized and purchased from GenScript (Piscataway, NJ, USA). Each peptide was dissolved in DMSO and stored at − 20 °C until use. For peptide transfection, the peptide was mixed with PULSin reagent (Polyplus Transfection, cat. #501-04, Illkirch-Graffenstaden, France) and added to a dish in which the cell medium had been replaced with fresh serum-free DMEM before transfection. The cell medium was replaced with fresh complete medium after 2 h.

### Immunoblotting and immunofluorescence staining

Cell lysates were quantified by Bradford assay. According to the detected protein size, SDS-PAGE gels at various percentages (8–13.5%) were used to separate proteins. For immunoblotting and immunofluorescence, antibodies against β-actin (Bioss, cat. #BS-0061R, Woburn, MA, USA), GAPDH (Millipore; cat. #MAB374, Burlington, MA, USA or Santa Cruz; cat. #sc-47724, Dallas, TX, USA), VDAC1 (Santa Cruz, cat. #sc-8828), MT-CO2 (Life Technologies, cat. #A6404, Carlsbad, CA, USA), p65 (Santa Cruz, cat. #sc-372 or sc-8008), phosphor-IRF3 (Cell Signaling, cat. #4947S, Danvers, MA, USA), ECSIT (Abcam, cat. #ab21288), MAVS (Santa Cruz, cat. #sc-166583), RIG-I (Santa Cruz, cat. #sc-376845), NDV HN (Santa Cruz, cat. #sc-53562), influenza A virus NP (Abcam, cat. #ab128193), TOM20 (Santa cruz, cat. #sc-17764) and HDAC1 (Santa Cruz, cat. #sc-6298) were used. For immunofluorescence staining, cells were seeded on cover glass that had been coated with poly-D-lysine. Then, the cells were fixed with 4% paraformaldehyde, permeabilized with 0.2% Triton X-100 and blocked with 1% BSA. An Alexa Fluor 488-conjugated secondary antibody (Invitrogen, cat. #A11017) was used in this study. To stain mitochondria, cells were incubated with 100–400 nM MitoTracker Deep Red (Invitrogen, cat. #M22426) in serum-free DMEM for 30 min. DAPI solution (Invitrogen, cat. #R37606) was used to visualize nuclei.

### Flow cytometry analysis

To measure mitochondrial membrane potential and mitochondrial ROS concentrations, cells were incubated with complete media containing 2.5 µM JC-1 dye (Thermo Fisher, cat. #T3168, Waltham, MA, USA) and HBSS (Gibco, cat. #14025-092, Carlsbad, CA, USA) containing 1–5 µM MitoSOX (Invitrogen, cat. #M36008), respectively. After a 30 min incubation at 37 °C in an incubator, the cells were harvested with FACS buffer (2% FBS in phosphate-buffered saline) and analyzed with a flow cytometer (BD Biosciences, San Jose, CA, USA).

### RNA isolation, quantitative real-time PCR and RNA sequencing

Total RNA was extracted from cultured cells using RNAiso Plus (Takara, cat. #9109, Kusatsu, Japan). cDNA synthesis was conducted using 500 ng of RNA with the ImProm-II Reverse Transcription System (Promega, cat. #A3803). To measure mRNA expression levels, quantitative PCR and real-time PCR were performed with a typical thermal cycler and a StepOnePlus Real-Time PCR system (Applied Biosystems, Waltham, MA, USA), respectively. RNA sequencing was performed with the Illumina NextSeq platform using a Library prep kit (TruSeq Stranded mRNA Sample Preparation kit; LAS Incorporated, Gimpo, Republic of Korea).

## Supplementary information

Supplementary file1
