# Supplementary material for: MTFMT deficiency correlates with reduced mitochondrial integrity and enhanced host susceptibility to intracellular infection
Source: Sci Rep. 2020 Jul 7;10:11183. doi: 10.1038/s41598-020-68053-8 (PMC7341849; doi:10.1038/s41598-020-68053-8)
Supplement: Supplementary file 1 — Supplementary file1 [file 41598_2020_68053_MOESM1_ESM.pdf]

MTFMT deficiency correlates with reduced mitochondrial integrity and enhanced host susceptibility to intracellular infection

Jung-Hwa Seo[1], Cheol-Sang Hwang[1], Joo-Yeon Yoo[1*]

[1] Department of Life Sciences, Pohang University of Science and Technology (POSTECH), Pohang 790-784, Republic of Korea

[*] Correspondence: jyoo@postech.ac.kr

a

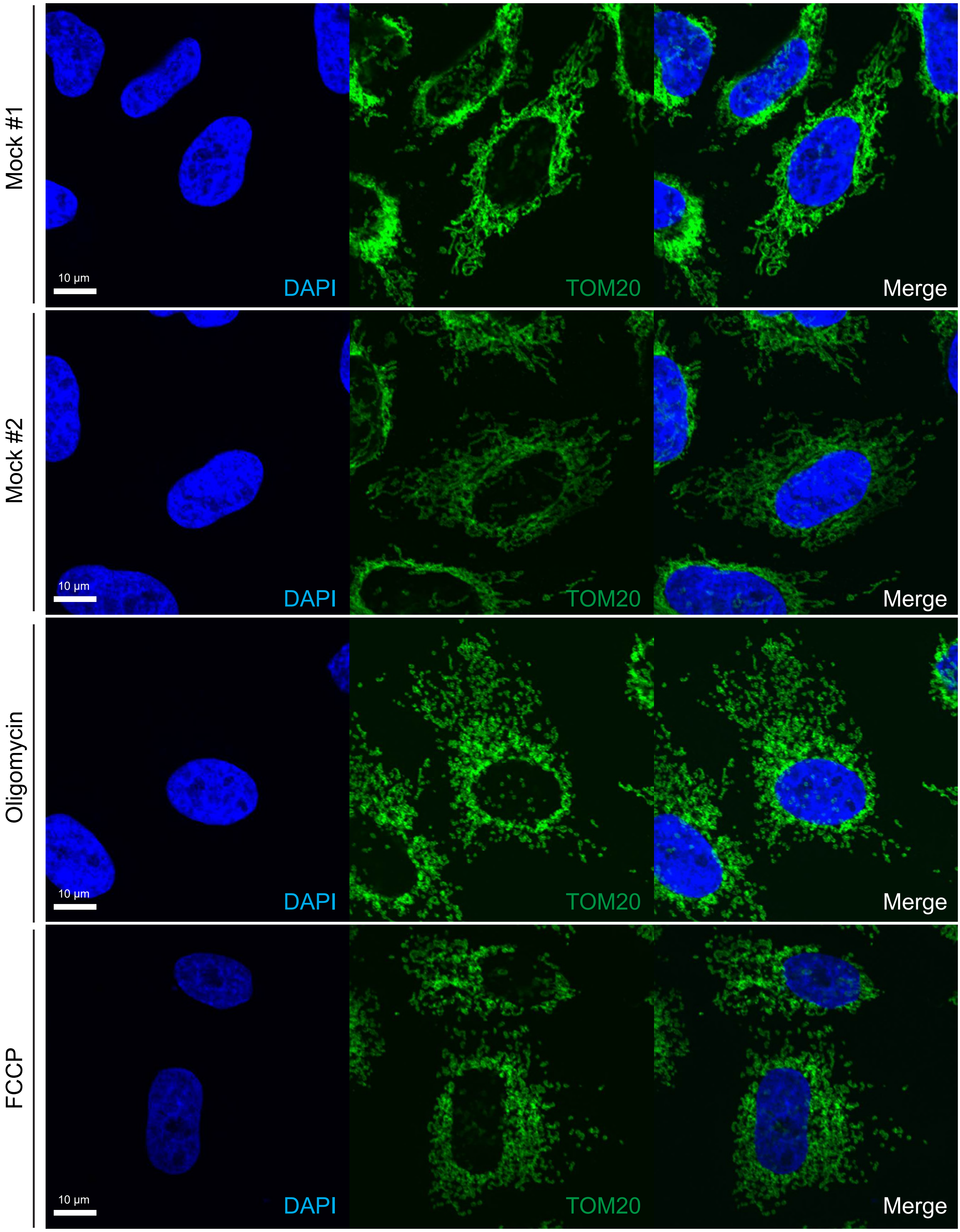

Mock #1
10 µm
DAPI
TOM20
Merge
Mock #2
10 µm
DAPI
TOM20
Merge
Oligomycin
10 µm
DAPI
TOM20
Merge
FCCP
10 µm
DAPI
TOM20
Merge

Supplementary Figure 1. Mitochondrial stresses induce mitochondrial fission.

(a) Control cells were treated with DMSO (first and second row). HeLa cells were treated with oligomycin (third row; concentration : 10 μM) or FCCP (forth row; concentration : 100 μM) for 3 hours and 15 min, respectively. Cells were stained with anti-TOM20 antibody, followed by anti-mouse Alexa 488. Nuclei were stained with DAPI.

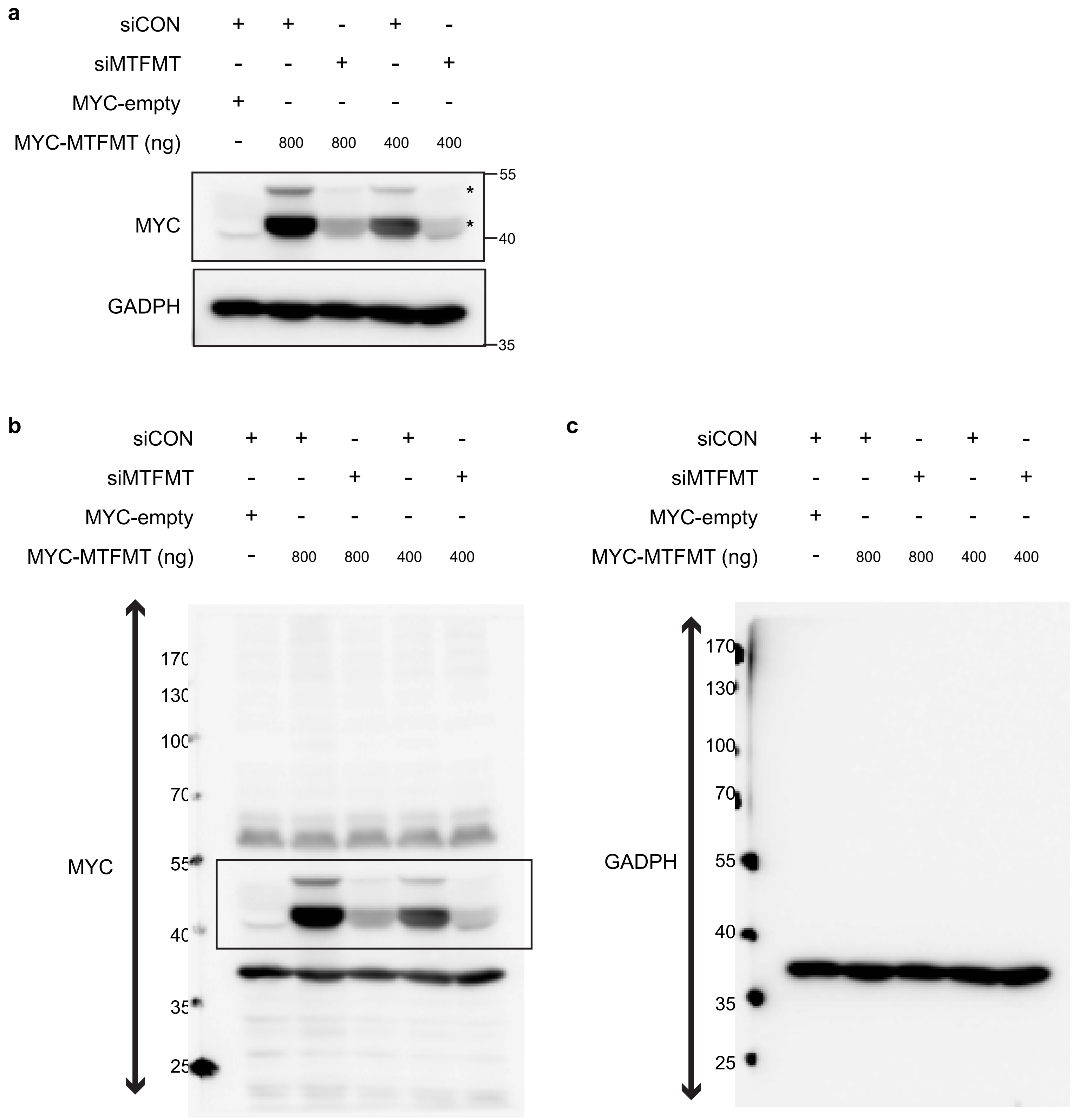
a
siCON + + - + -
siMTFMT - - + - +
MYC-empty + - - - -
MYC-MTFMT (ng) - 800 800 400 400
MYC
*
*
55
40
GADPH
35
b
siCON + + - + -
siMTFMT - - + - +
MYC-empty + - - - -
MYC-MTFMT (ng) - 800 800 400 400
MYC
170
130
100
70
55
40
35
25
c
siCON + + - + -
siMTFMT - - + - +
MYC-empty + - - - -
MYC-MTFMT (ng) - 800 800 400 400
GADPH
170
130
100
70
55
40
35
25

Supplementary Figure 2. Knock-down efficiency of MTFMT siRNA is confirmed with MTFMT protein level.

(a) HeLa cells were co-transfected with MYC-tagged MTFMT and control or MTFMT siRNA. 48 hours after transfection, cells were lysed and examined for immunoblotting assay. Membrane was blotted with anti-MYC antibody to detect exogenous MTFMT.

(b, c) Full blot image of membrane from a. After stripping of membrane, membrane was blotted again with anti-GAPDH antibody (c). Black boxes were cropped and presented in a.

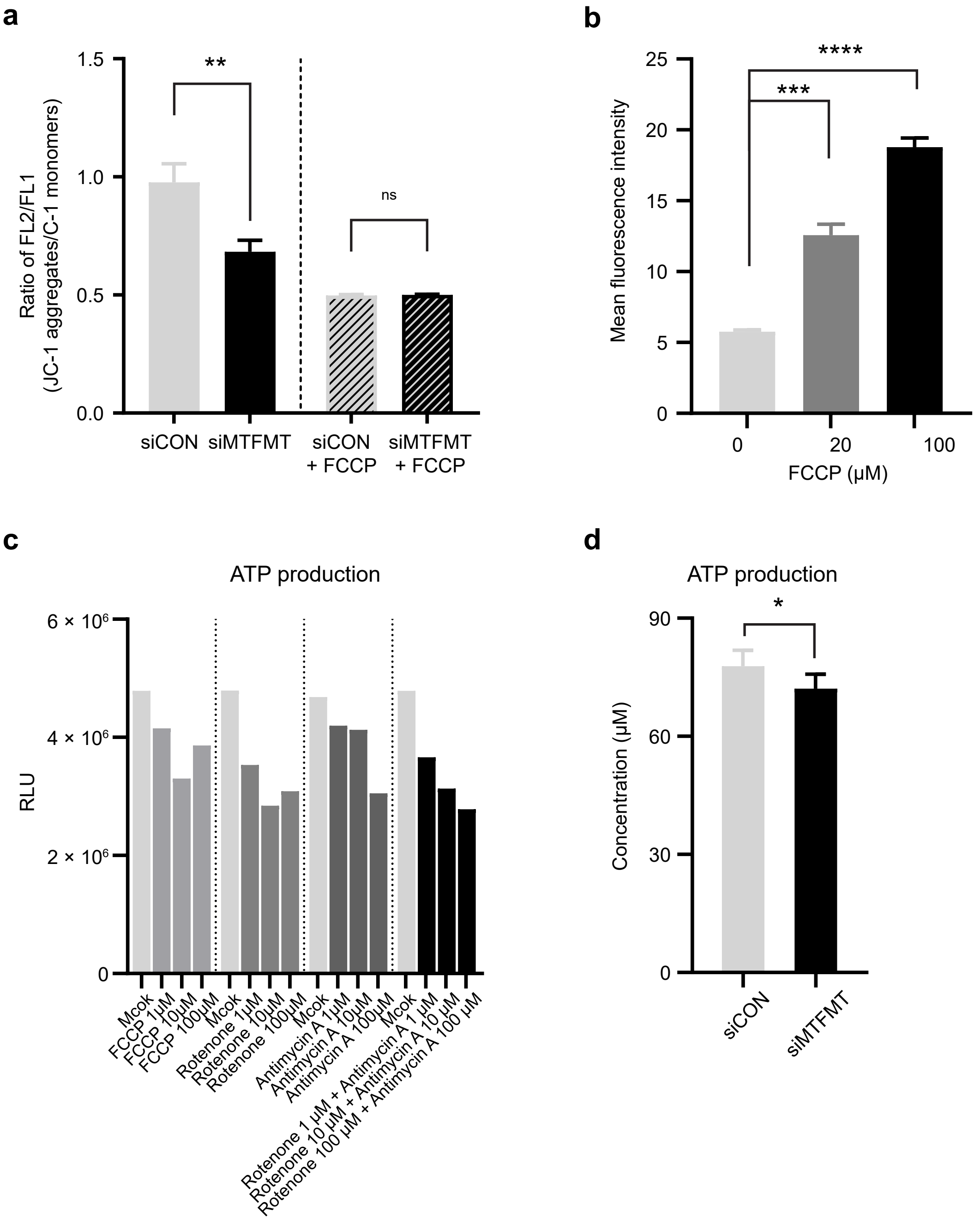

a
Ratio of FL2/FL1
(JC-1 aggregates/C-1 monomers)
1.5
1.0
0.5
0.0
**
ns
siCON
siMTFMT
siCON
+ FCCP
siMTFMT
+ FCCP
b
Mean fluorescence intensity
25
20
15
10
5
0
****
***
0
20
100
FCCP (μM)
c
ATP production
RLU
6 × 10⁶
4 × 10⁶
2 × 10⁶
0
Mcok
FCCP 1μM
FCCP 10μM
FCCP 100μM
Mcok
Rotenone 1μM
Rotenone 10μM
Rotenone 100μM
Mcok
Antimycin A 1μM
Antimycin A 10μM
Antimycin A 100μM
Mcok
Rotenone 1 μM + Antimycin A 1 μM
Rotenone 10 μM + Antimycin A 10 μM
Rotenone 100 μM + Antimycin A 100 μM
d
ATP production
Concentration (μM)
90
60
30
0
*
siCON
siMTFMT

Supplementary Figure 3. MTFMT deficiency induces loss of mitochondrial membrane potential and ATP production.

(a) Forty-eight hours after siRNA transfection, cell medium was replaced with fresh medium containing 2.5 μM JC-1 dye with or without 50 μM FCCP and incubated for 15 min at 37°C. Fluorescence intensity was analyzed using FACS. Error bars indicate SDs and statistical significance was assessed by unpaired t-test. **, P < 0.01.

(b) After treatment of indicated concentration of FCCP, HeLa cells were stained with 1 μM MitoSOX for 30 min at 37°C and checked for fluorescence intensity using FACS. Error bars indicate SDs and statistical significance was assessed by unpaired t-test. ***, P < 0.001; ****, P < 0.0001.

(c) HeLa cells were treated with FCCP, rotenone, antimycin A or mixture of rotenone and antimycin A (at the following concentrations : 1, 10 and 100 μM) for 6 hours. After washing cells with PBS, cells were lysed with 1X Passive lysis buffer and examined for ATP concentration within cells. See Materials and Methods for detailed protocol.

(d) Forty-eight hours after siRNA transfection, cellular ATP concentration was measured using luminescent ATP detection assay. See Materials and Methods for detailed protocol. According to ATP standard curve, actual ATP concentration was calculated. Error bars indicate SDs and statistical significance was assessed by unpaired t-test. *, P < 0.05

a

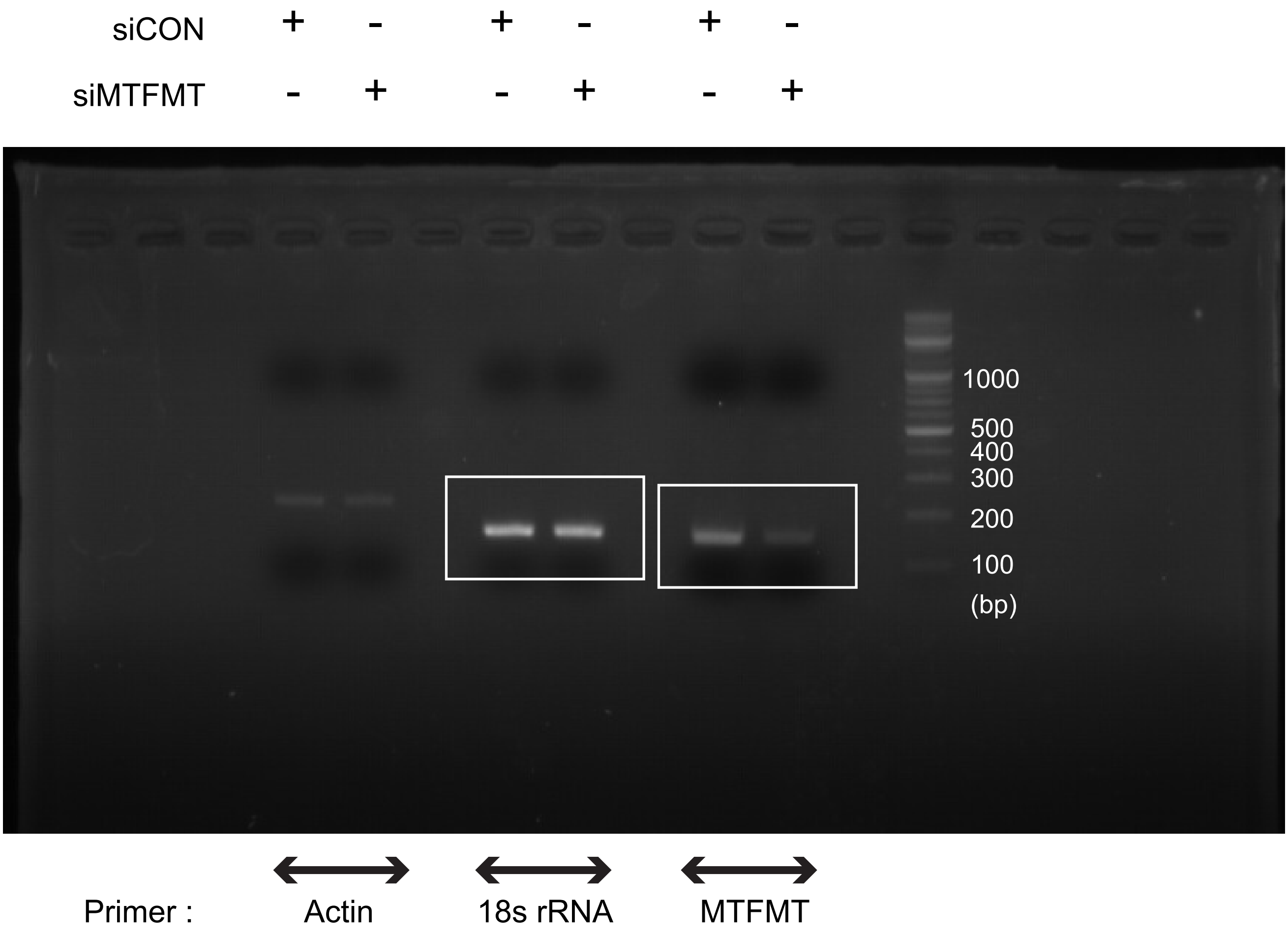
siCON + - + - + -
siMTFMT - + - + - +
1000
500
400
300
200
100
(bp)
Primer :
Actin
18s rRNA
MTFMT

Supplementary Figure 4. The MTFMT knockdown efficiency is assessed by RT-PCR.

(a) HeLa cells transfected with control or MTFMT were harvested for RT-PCR. The rest of cells were stained with JC-1 or MitoSOX as described in Materials and Methods. RT-PCR products were run on 2% agarose gel. After inversion of gel image, white boxes were cropped and presented in Fig. 1b. Ladder is 100 bp plus DNA ladder (Bioneer). Samples are listed at the top and primers are listed at the bottom.

a
b

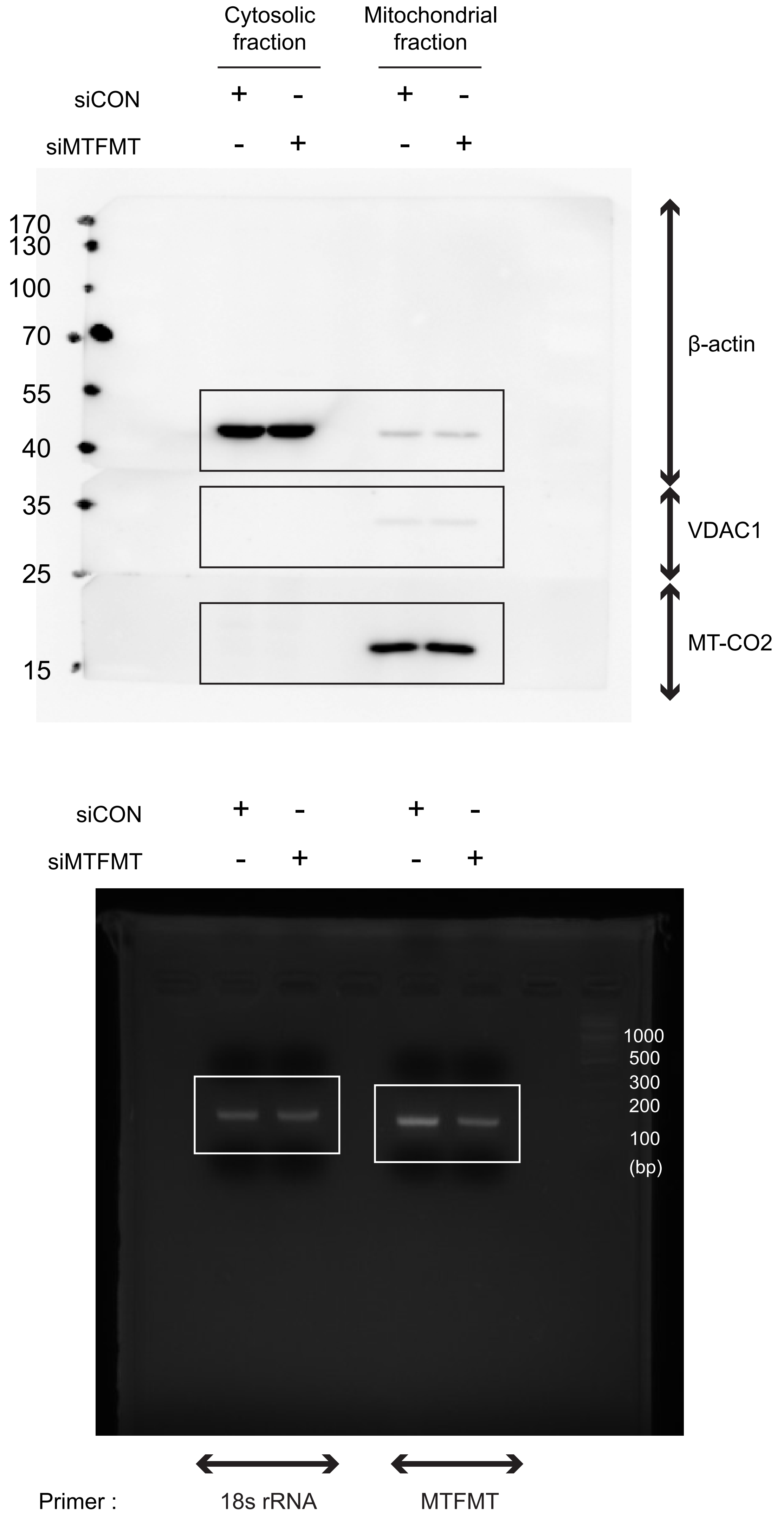

Cytosolic fraction
Mitochondrial fraction
siCON + - + -
siMTFMT - + - +
170
130
100
70
55
40
35
25
15
β-actin
VDAC1
MT-CO2
siCON + - + -
siMTFMT - + - +
1000
500
300
200
100
(bp)
Primer :
18s rRNA
MTFMT

Supplementary Figure 5. Mitochondrial translational efficiency is examined by western blot assay and related MTFMT knockdown efficiency is assessed by RT-PCR.

(a, b) HeLa cells transfected with control or MTFMT were harvested for western blot (a) and RT-PCR (b). After cell fractionation as described in Materials and Methods, samples were run on 10% polyacrylamide gel (a). Black boxes were cropped and presented in Fig. 1e (left). Samples are listed at the top and antibodies are listed at right. RT-PCR products were run on 2% agarose gel (b). After inversion of gel image, white boxes were cropped and presented in Fig. 1e (right). Samples are listed at the top and primers are listed at the bottom.

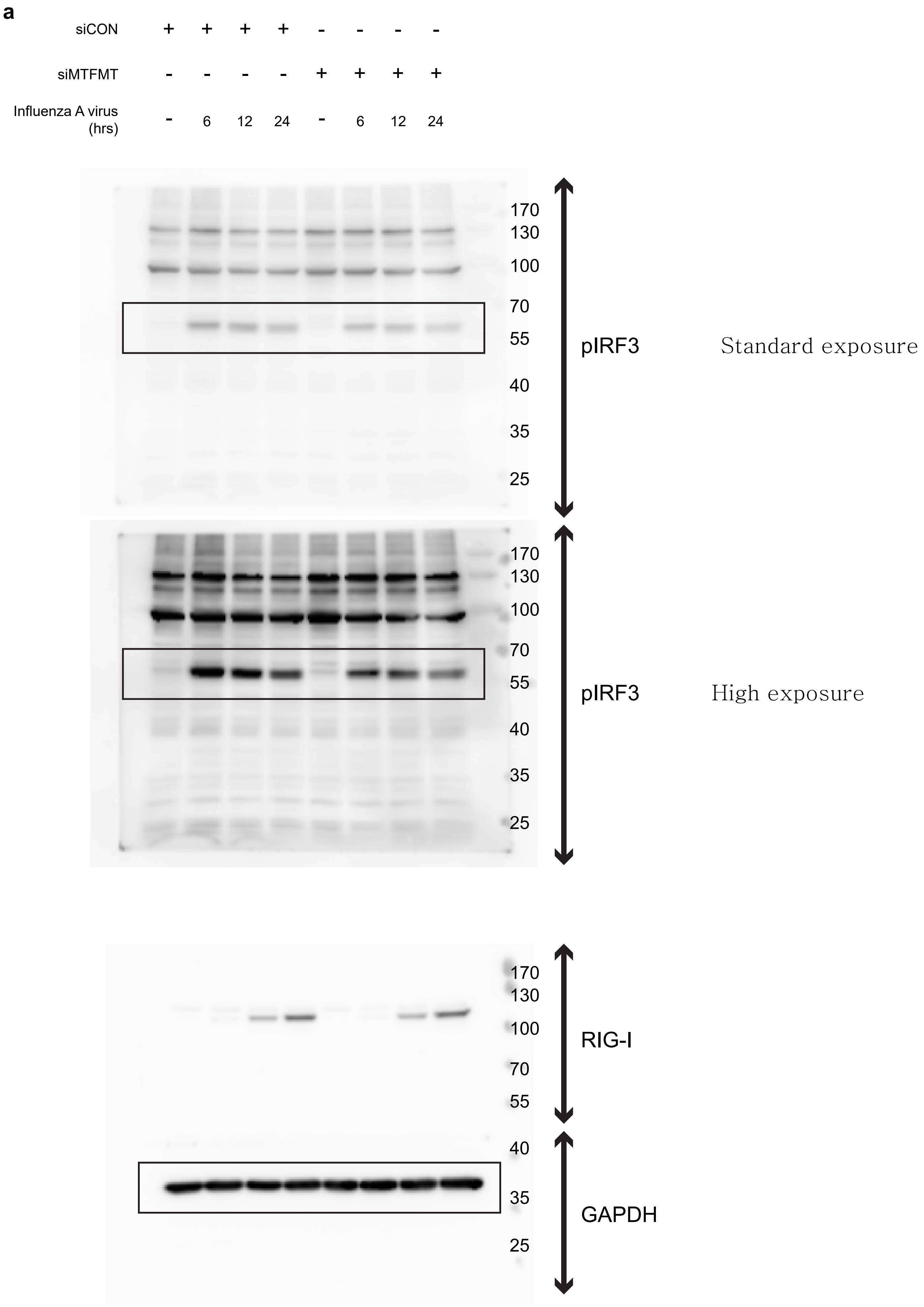
a
siCON
siMTFMT
Influenza A virus (hrs)
- 6 12 24 - 6 12 24
170
130
100
70
55
40
35
25
pIRF3
Standard exposure
High exposure
RIG-I
GAPDH

b

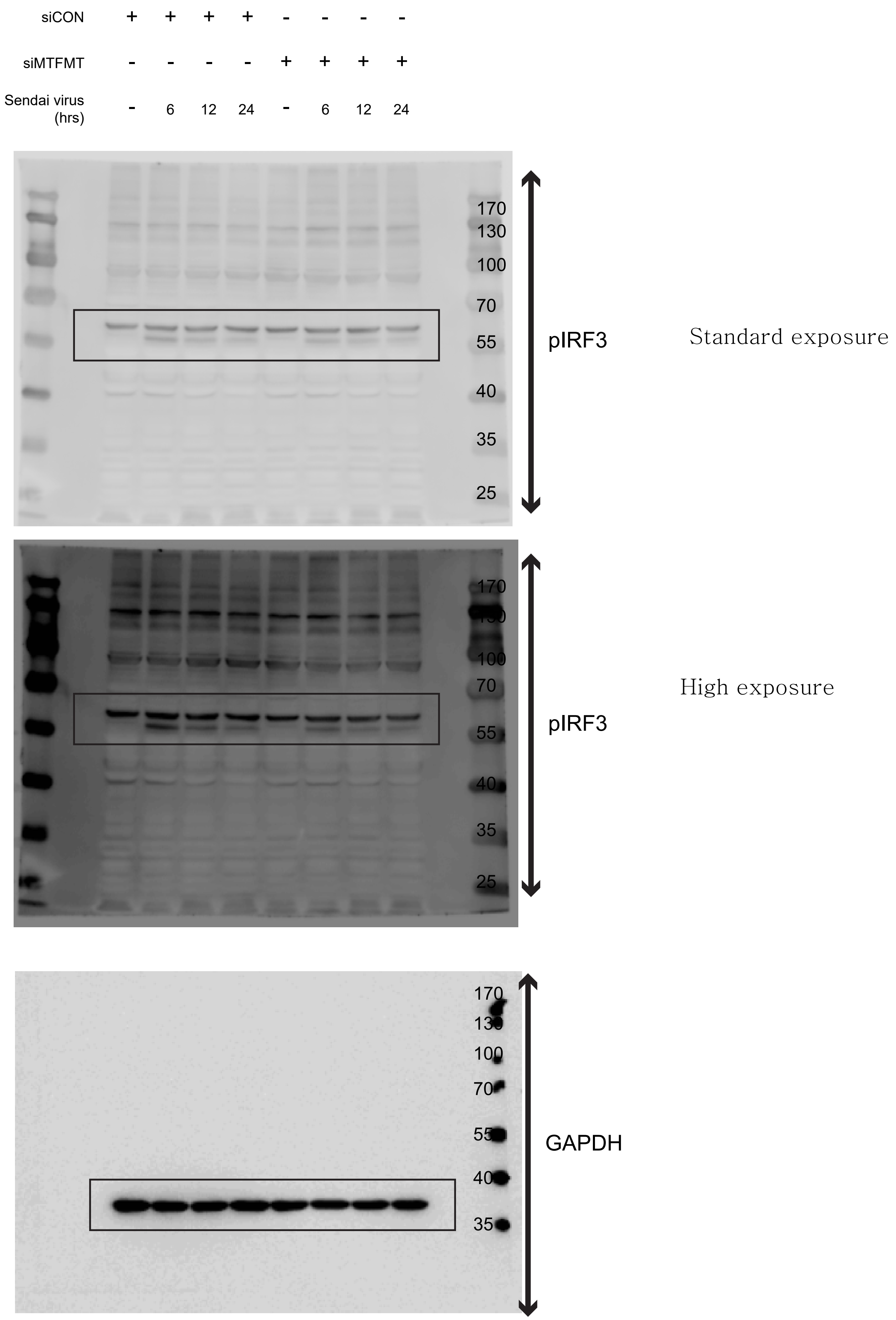
siCON + + + + - - - -
siMTFMT - - - - + + + +
Sendai virus (hrs) - 6 12 24 - 6 12 24
170
130
100
70
55
40
35
25
pIRF3
Standard exposure
High exposure
GAPDH

c

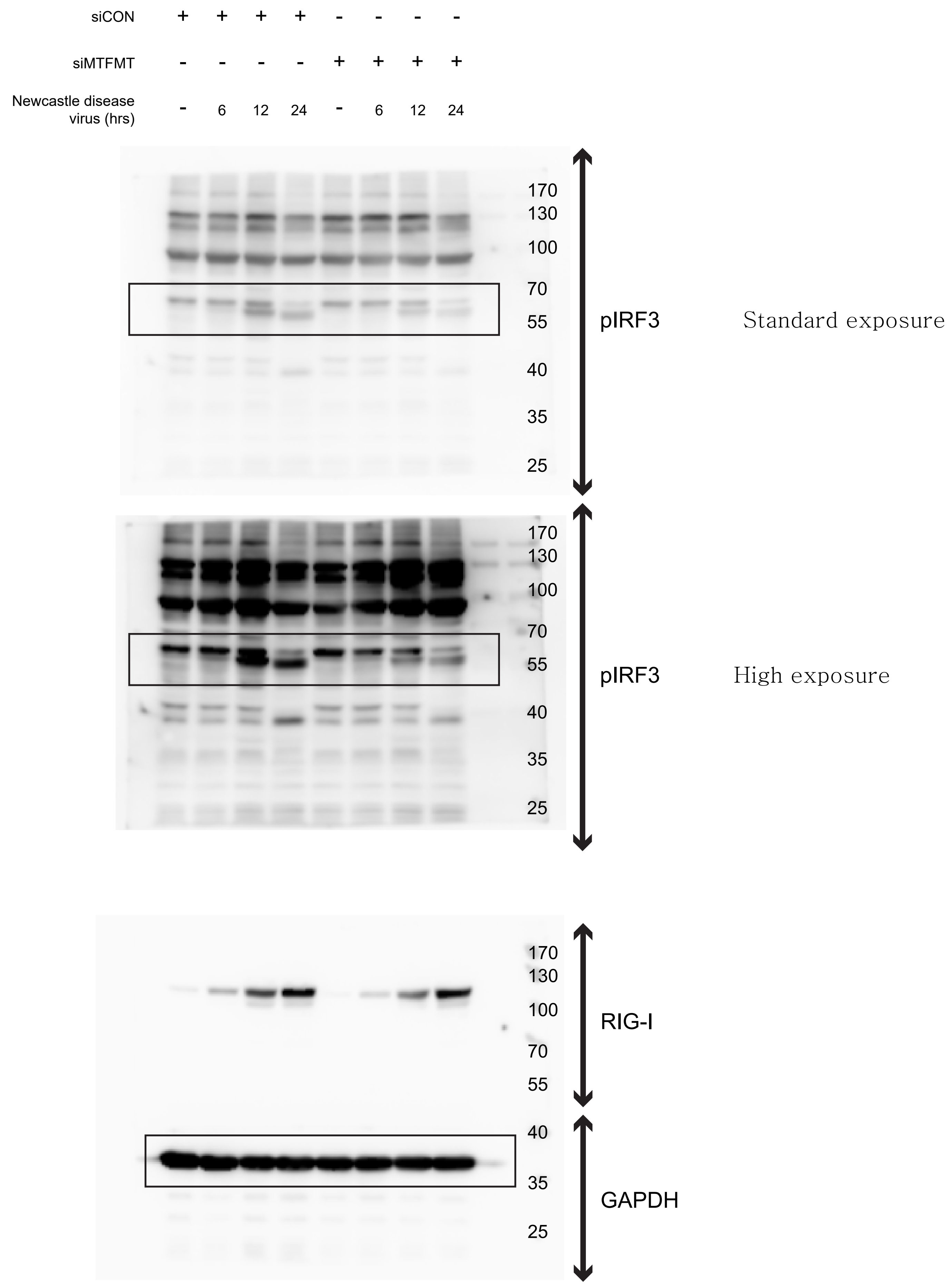
siCON + + + + - - - -
siMTFMT - - - - + + + +
Newcastle disease virus (hrs) - 6 12 24 - 6 12 24
170
130
100
70
55
40
35
25
pIRF3
Standard exposure
170
130
100
70
55
40
35
25
pIRF3
High exposure
170
130
100
70
55
RIG-I
40
35
25
GAPDH

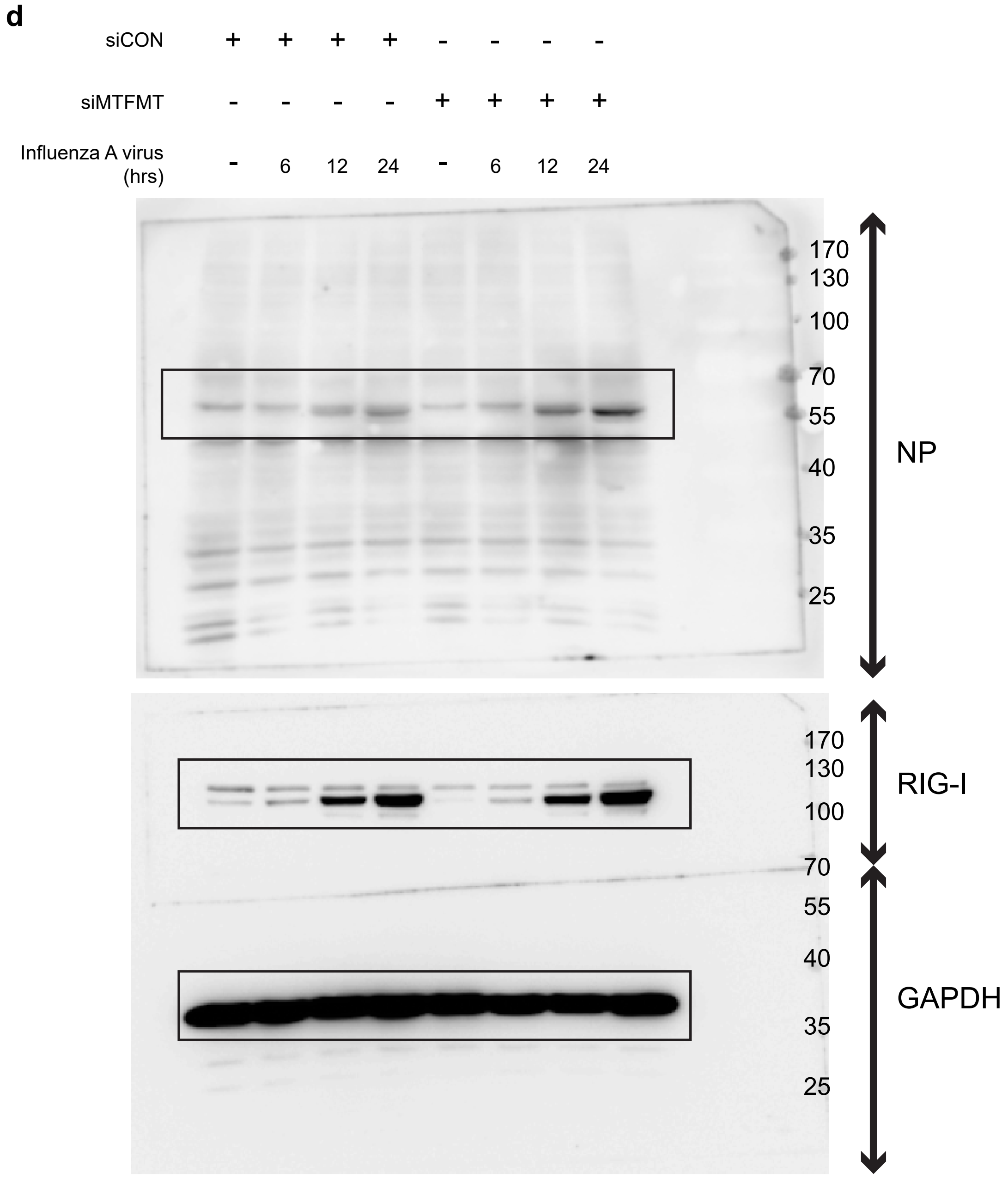
d
siCON
siMTFMT
Influenza A virus (hrs)
- 6 12 24 - 6 12 24
170
130
100
70
55
40
35
25
NP
RIG-I
GAPDH

Supplementary Figure 6. MTFMT deficiency enhances viral replication by inhibiting RIG-I-mediated signaling pathway.

(a) Full blot image of membrane from Fig. 2g (left). Samples are listed at the top and antibodies are listed at right. After stripping of membrane, membrane was blotted again with an antibody listed at right (bottom). Black boxes were cropped and presented in Fig. 2g, left.

(b) Full blot image of membrane from Fig. 2g (middle). After stripping of membrane, membrane was blotted again with antibodies listed at right (bottom). Black boxes were cropped and presented in Fig. 2g, middle.

(c) Full blot image of membrane from Fig. 2g (right). After stripping of membrane, membrane was blotted again with antibodies listed at right (bottom). Black boxes were cropped and presented in Fig. 2g, right.

(d) Full blot image of membrane from Fig. 2h. After stripping of membrane, membrane was blotted again with antibody listed at right (bottom). Black boxes were cropped and presented in Fig. 2h.

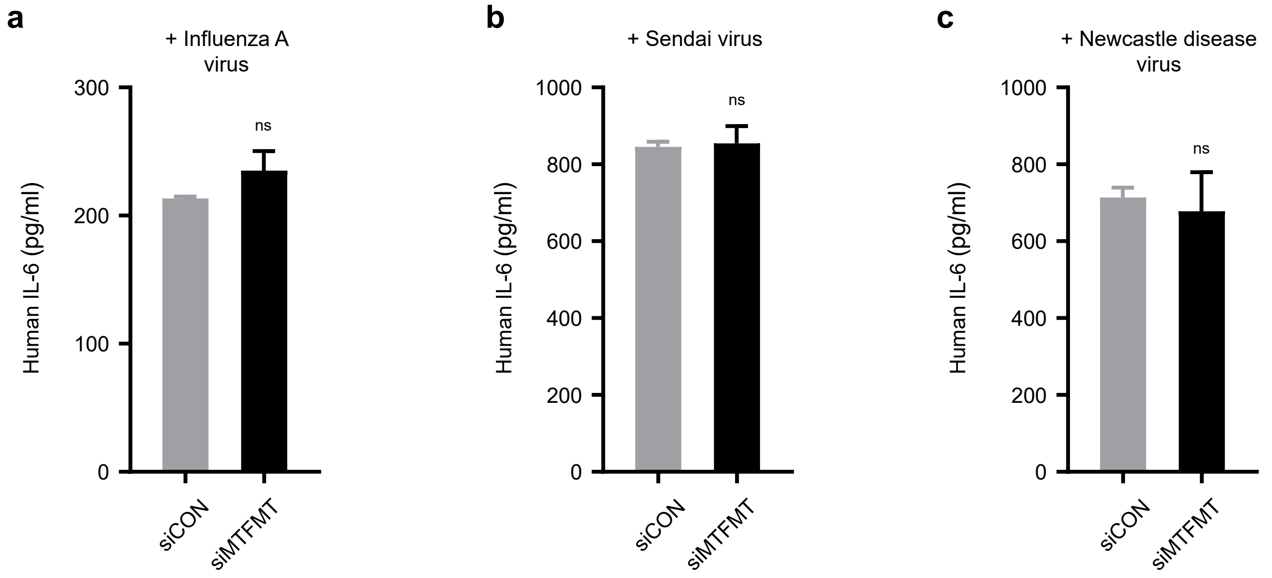
a
+ Influenza A virus
Human IL-6 (pg/ml)
300
200
100
0
ns
siCON
siMTFMT
b
+ Sendai virus
Human IL-6 (pg/ml)
1000
800
600
400
200
0
ns
siCON
siMTFMT
c
+ Newcastle disease virus
Human IL-6 (pg/ml)
1000
800
600
400
200
0
ns
siCON
siMTFMT

Supplementary Figure 7. MTFMT deficiency does not affect a production of IL-6 upon RNA virus infections.

(a-c) Forty-eight hours after siRNA transfection, HeLa cells were infected with influenza A virus (a), Sendai virus (b), or Newcastle disease virus (c) for 12 hours. Cell medium were harvested for quantifying human IL-6 concentration as described in Materials and Methods. Error bars of graphs from a-c indicate SDs and statistical significances were assessed by unpaired t-test.

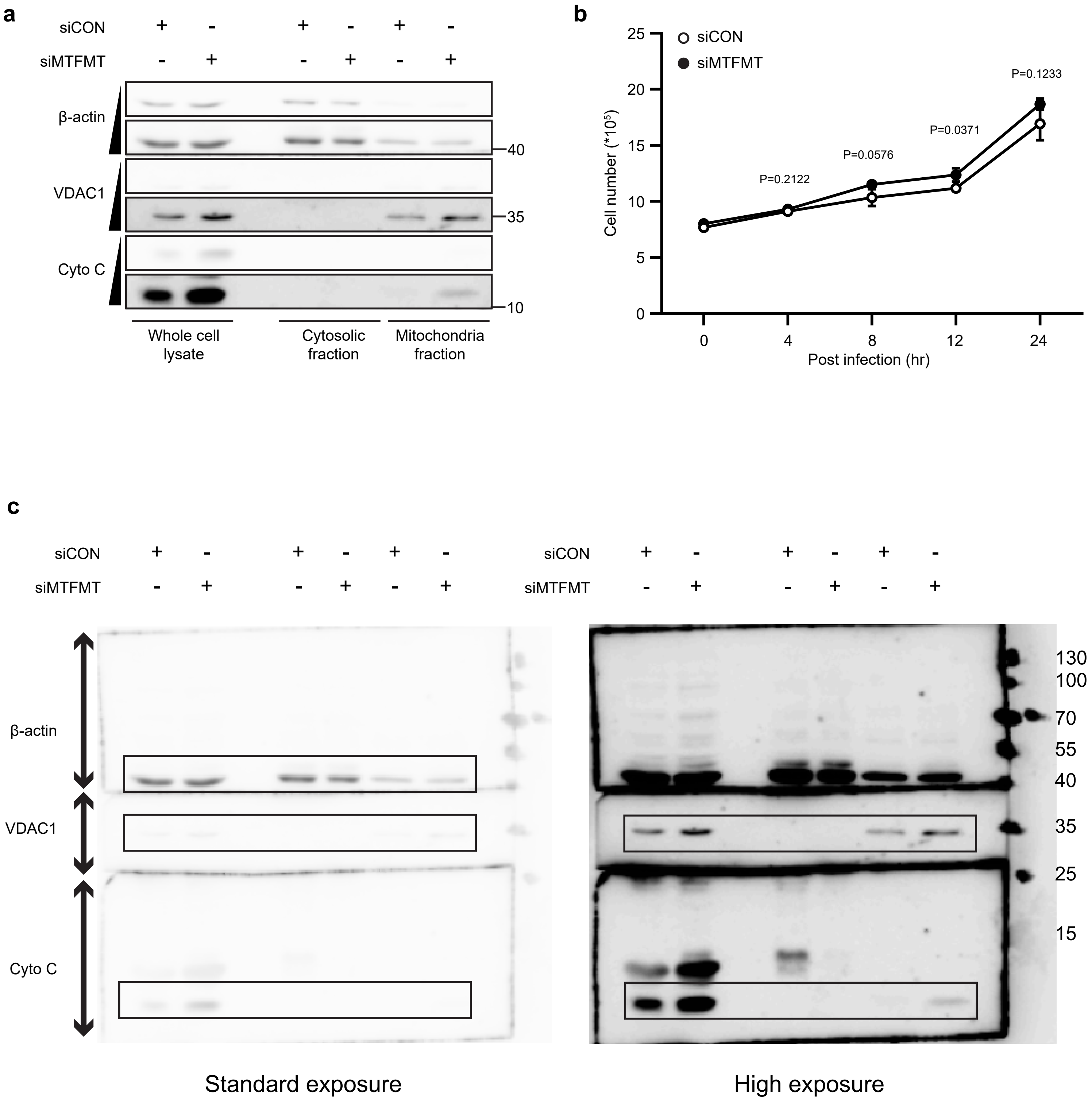
a
siCON
siMTFMT
β-actin
VDAC1
Cyto C
40
35
10
Whole cell lysate
Cytosolic fraction
Mitochondria fraction
b
siCON
siMTFMT
Cell number (*10⁵)
Post infection (hr)
P=0.2122
P=0.0576
P=0.0371
P=0.1233
0
4
8
12
24
c
siCON
siMTFMT
β-actin
VDAC1
Cyto C
130
100
70
55
40
35
25
15
Standard exposure
High exposure

Supplementary Figure 8. MTFMT deficiency does not induce cell death.

(a) HeLa cells were fractionated to cytosolic- and mitochondrial fraction as described in Materials and Methods. In order to check induction of programmed cell death, cytosolic levels of cytochrome C protein were measured by immunoblotting assay.

(b) Forty-eight hours after siRNA transfection, HeLa cells were infected with S. flexneri (MOI=10) for 24 hours. In order to check induction of cell death, live cell numbers were counted using a hemocytometer at indicated time points. Error bars indicate SDs and statistical significance was assessed by unpaired t-test.

(c) Full blot image of membrane from a. Black boxes were cropped and presented in a.

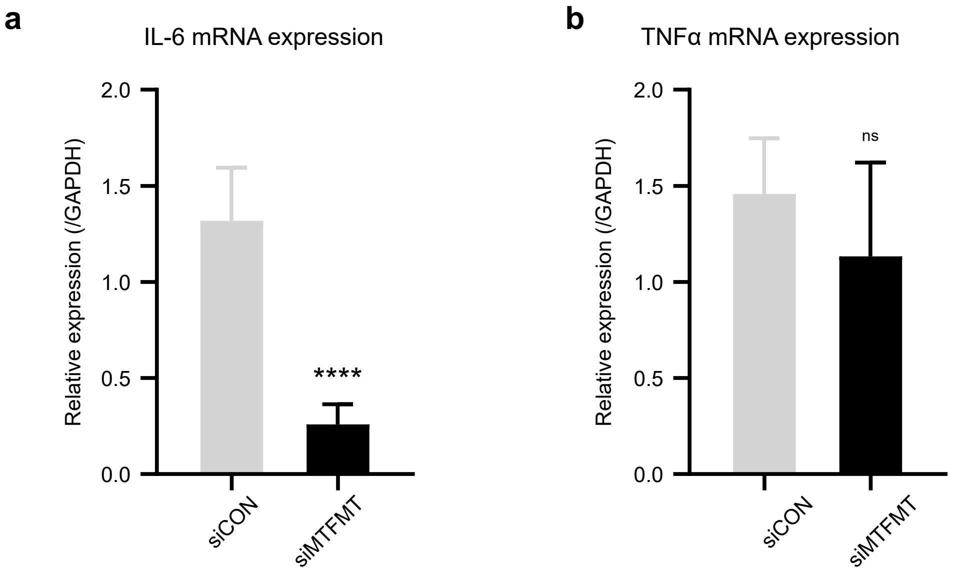
a
IL-6 mRNA expression
Relative expression (/GAPDH)
2.0
1.5
1.0
0.5
0.0
****
siCON
siMTFMT
b
TNFα mRNA expression
Relative expression (/GAPDH)
2.0
1.5
1.0
0.5
0.0
ns
siCON
siMTFMT

Supplementary Figure 9. MTFMT silencing decrease mRNA expression level of IL-6, but not TNFα.

(a, b) HeLa cells transfected with control or MTFMT siRNA were harvested for real-time PCR analysis. Specific primers which detect IL-6 mRNA (a) or TNFα mRNA (b) were used for amplification. Error bars indicate SDs and statistical significance was assessed by unpaired t-test. ****, $P < 0.0001$.

a

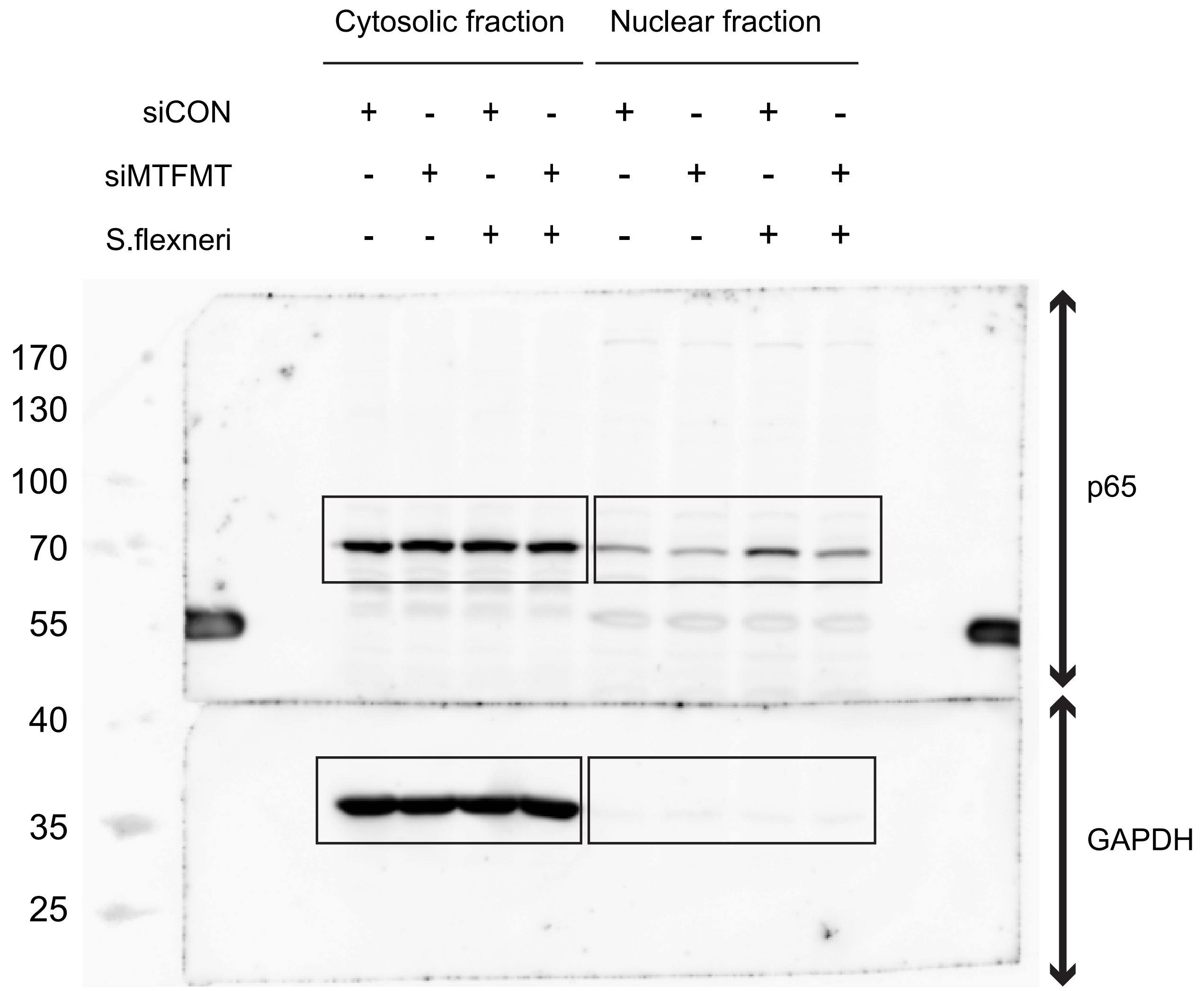
Cytosolic fraction
Nuclear fraction
siCON + - + - + - + -
siMTFMT - + - + - + - +
S.flexneri - - + + - - + +
170
130
100
70
55
40
35
25
p65
GAPDH

b

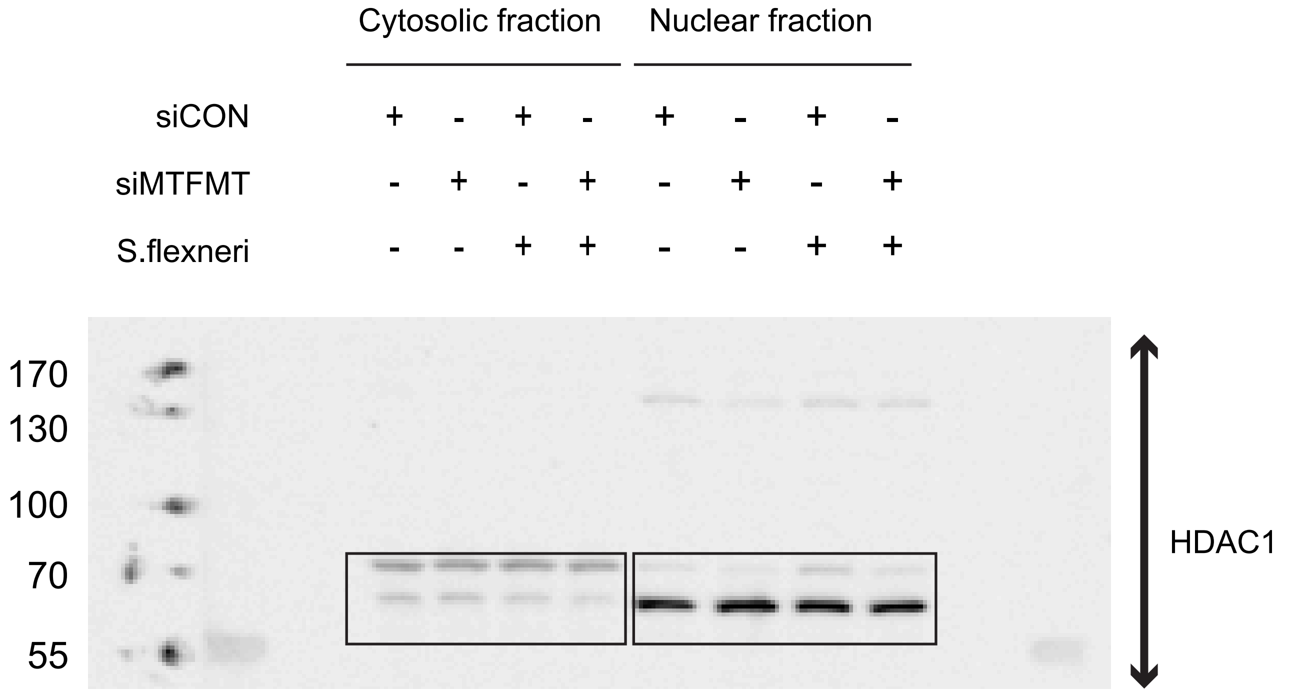
Cytosolic fraction
Nuclear fraction
siCON + - + - + - + -
siMTFMT - + - + - + - +
S.flexneri - - + + - - + +
170
130
100
70
55
HDAC1

Supplementary Figure 10. MTFMT deficiency inhibits nuclear translocation of p65.

(a, b) Full blot image of membrane from Fig. 4f. After stripping of upper membrane, membrane was blotted again with an antibody listed at right (b). Black boxes were cropped and presented in Fig. 4f.

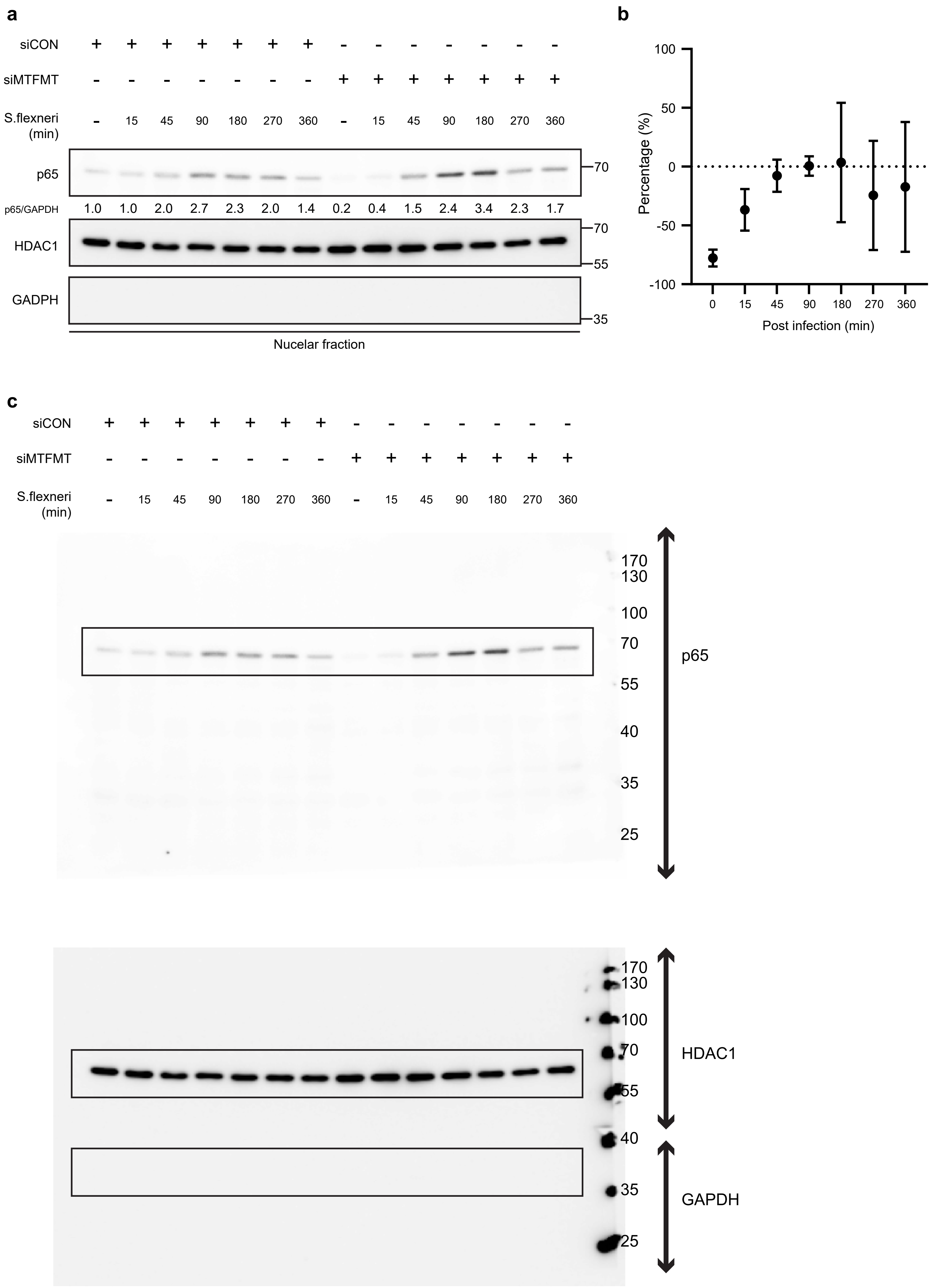
a
siCON
siMTFMT
S.flexneri (min)
15 45 90 180 270 360
p65
p65/GAPDH
1.0 1.0 2.0 2.7 2.3 2.0 1.4 0.2 0.4 1.5 2.4 3.4 2.3 1.7
HDAC1
GADPH
70
55
35
Nucelar fraction
b
Percentage (%)
100
50
0
-50
-100
0 15 45 90 180 270 360
Post infection (min)
c
siCON
siMTFMT
S.flexneri (min)
15 45 90 180 270 360
170
130
100
70
55
40
35
25
p65
HDAC1
GAPDH

Supplementary Figure 11. MTFMT deficiency inhibits nuclear translocation of p65 responding to *Shigella flexneri* infection.

(a, b) Nuclear fractions of HeLa cells transfected with control or MTFMT siRNA were blotted for p65 before and after *S. flexneri* infection (MOI=100) (a). The relative change of band intensity of p65 between control- and MTFMT siRNA-transfected cells was shown in b.

(c) Full blot image of membrane from a. After stripping of upper membrane, membrane was blotted again with antibodies listed on right (bottom). Black boxes were cropped and presented in a.

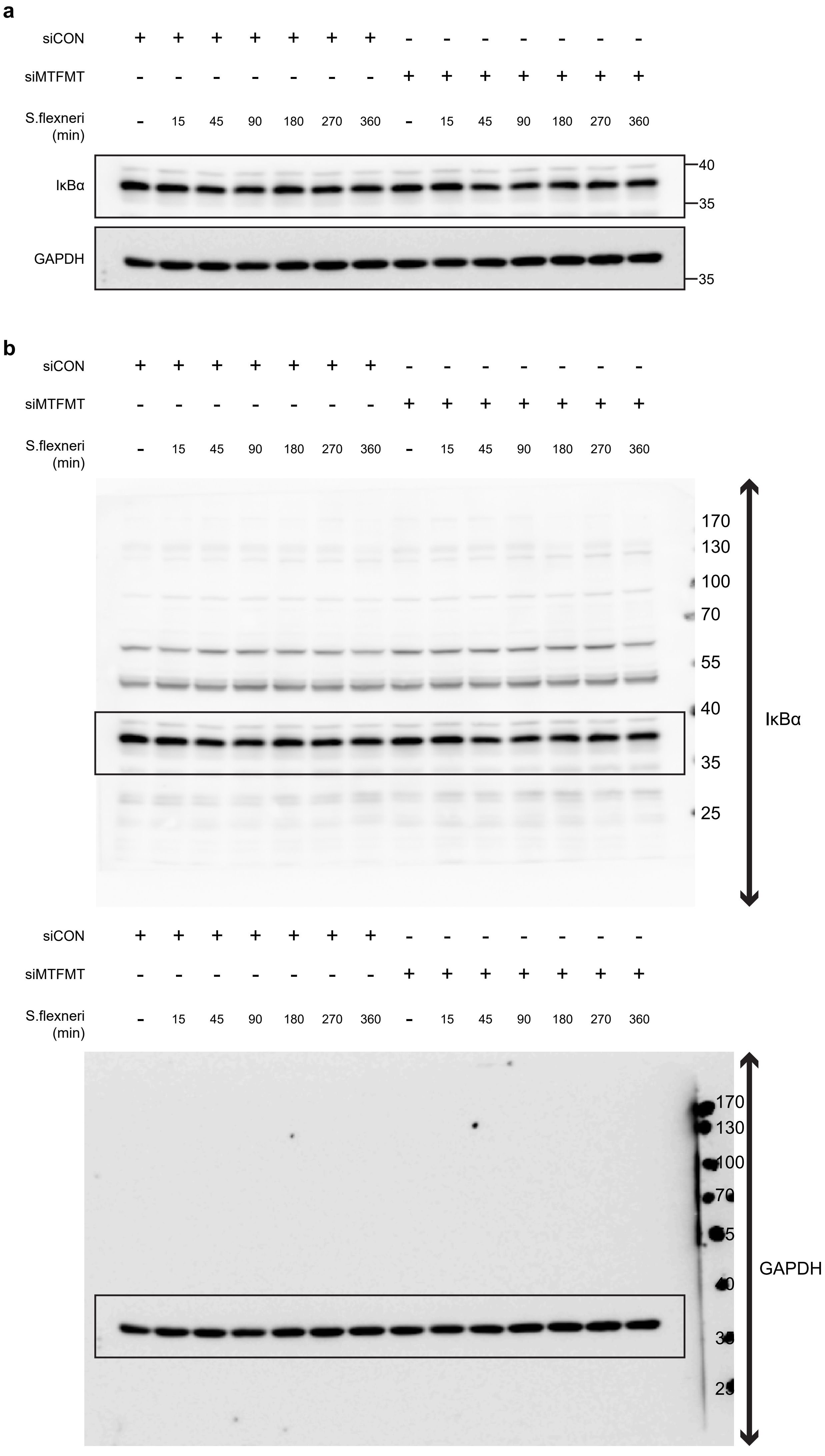
a
siCON
siMTFMT
S.flexneri (min)
15
45
90
180
270
360
IκBα
GAPDH
40
35
b
170
130
100
70
55
25
GAPDH

Supplementary Figure 12. MTFMT deficiency does not affect IκBα degradation during *Shigella flexneri* infection.

(a) HeLa cells transfected with control or MTFMT siRNA were harvested for immunoblotting assay after *S. flexneri* infection (MOI=100) at indicated time points.

(b) Full blot image of membrane from a. After stripping of upper membrane, membrane was blotted again with anti-GAPDH antibody (bottom). Black boxes were cropped and presented in a.

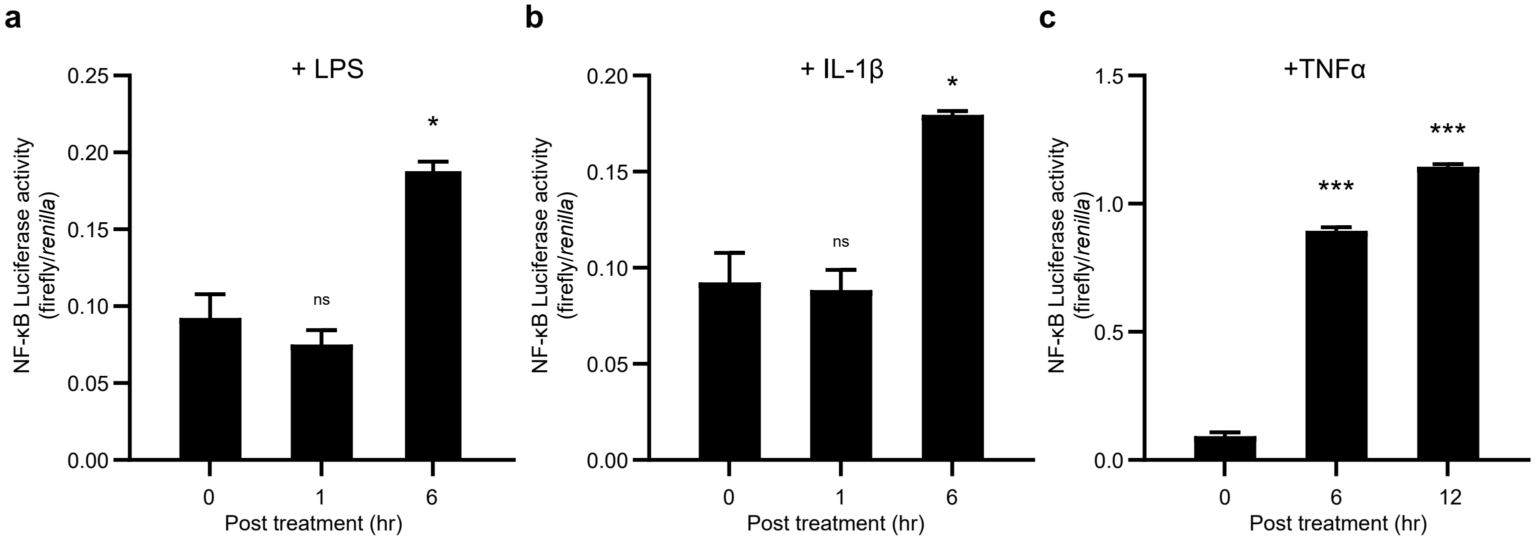
a
+ LPS
NF-κB Luciferase activity (firefly/renilla)
0.25
0.20
0.15
0.10
0.05
0.00
ns
*
0
1
6
Post treatment (hr)
b
+ IL-1β
NF-κB Luciferase activity (firefly/renilla)
0.20
0.15
0.10
0.05
0.00
ns
*
0
1
6
Post treatment (hr)
c
+TNFα
NF-κB Luciferase activity (firefly/renilla)
1.5
1.0
0.5
0.0
***
***
0
6
12
Post treatment (hr)

Supplementary Figure 13. Positive controls of NF-κB luciferase construct are tested.

(a-c) Forty-eight hours after co-transfection of pLuc-NF-κB with p*Renilla*-GAPDH, HeLa cells were treated with LPS (5 μg/ml), human IL-1β (25 ng/ml), and human TNFα (20 ng/ml) for indicated time points. Luciferase activities were measured by dual-luciferase assay as described in Materials and Methods. Error bars of graphs from a-c indicate SDs and statistical significance was assessed by unpaired t-test. *, $P < 0.05$; ***, $P < 0.001$.

a

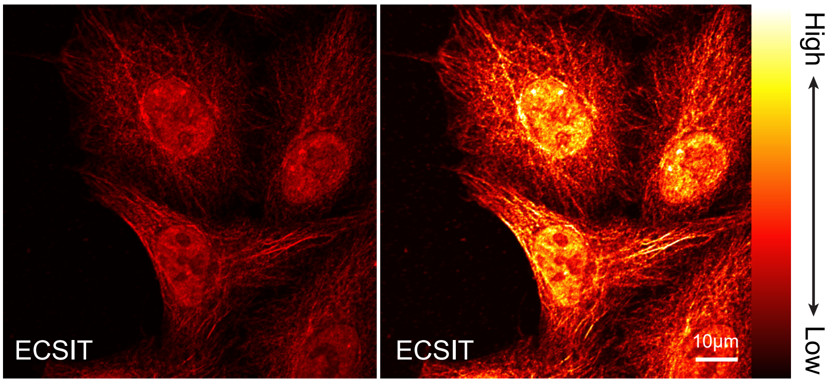

Supplementary Figure 14. Endogenous ECSIT protein distribution in HeLa.

(a) HeLa cells were stained for endogenous ECSIT, and representative confocal microscopy image is shown (left). Fluorescence intensity from left image is pseudo-colored, where white indicates higher ECSIT distribution.

a

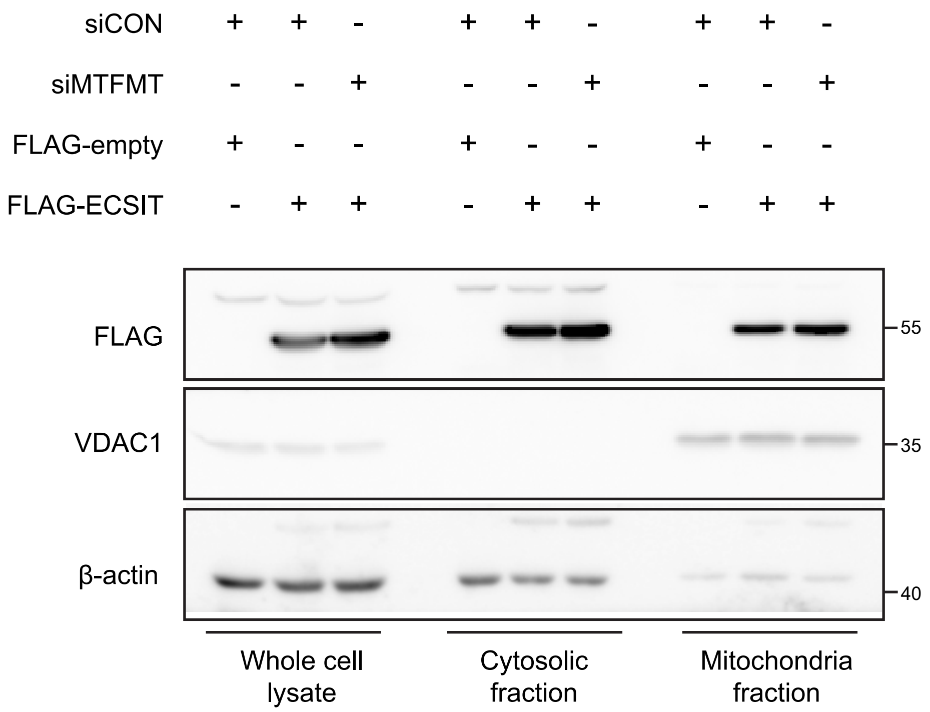

siCON + + - + + - + + -
siMTFMT - - + - - + - - +
FLAG-empty + - - + - - + - -
FLAG-ECSIT - + + - + + - + +
FLAG
VDAC1
β-actin
55
35
40
Whole cell lysate
Cytosolic fraction
Mitochondria fraction

b

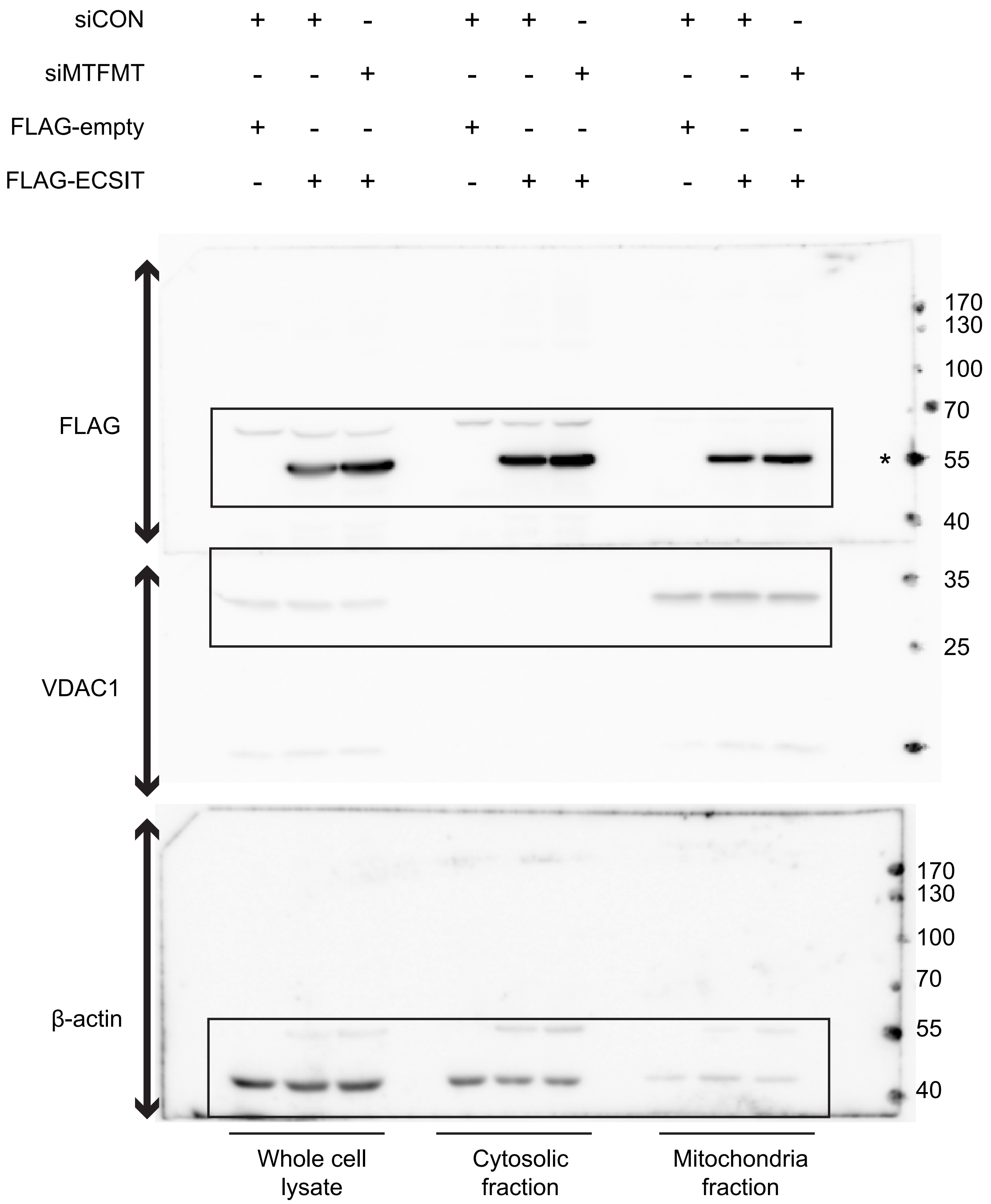

siCON + + - + + - + + -
siMTFMT - - + - - + - - +
FLAG-empty + - - + - - + - -
FLAG-ECSIT - + + - + + - + +
FLAG
VDAC1
β-actin
*
170
130
100
70
55
40
35
25
170
130
100
70
55
40
Whole cell lysate
Cytosolic fraction
Mitochondria fraction

Supplementary Figure 15. MTFMT silencing slightly increases a protein level of exogenous ECSIT in mitochondria.

(a) HeLa cells were co-transfected with FLAG-tagged ECIST and control or MTFMT siRNA. Forty-eight hours after transfection, cells were fractionated as described in Materials and Methods and each fraction was prepared for immunoblotting assay. To measure the protein level of exogenous ECIST, samples were run on 9% polyacrylamide gel and membrane was incubated with anti-FLAG antibody.

(b) Full blot image of membrane from a. After stripping of upper membrane, membrane was blotted again with anti-β-actin antibody. Black boxes were cropped and presented in a.

a

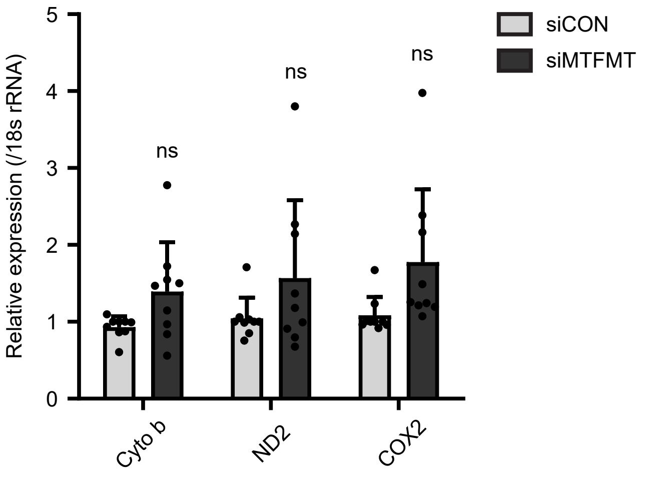

Relative expression (/18s rRNA)
0
1
2
3
4
5
ns
ns
ns
Cyto b
ND2
COX2
siCON
siMTFMT

Supplementary Figure 16. Concentration of cytosolic mtDNA is not changed by MTFMT deficiency.

(a) HeLa cells transfected with control or MTFMT siRNA were fractionated as described in Materials and Methods. To quantify cytoplasmic mtDNA copy number, total DNA was isolated from cytosolic fraction using DNeasy Blood and Tissue Kit (Qiagen), followed by amplifications of three types of mtDNA by Real-time qPCR. Error bar indicates SD obtained from three independent experiments.

a

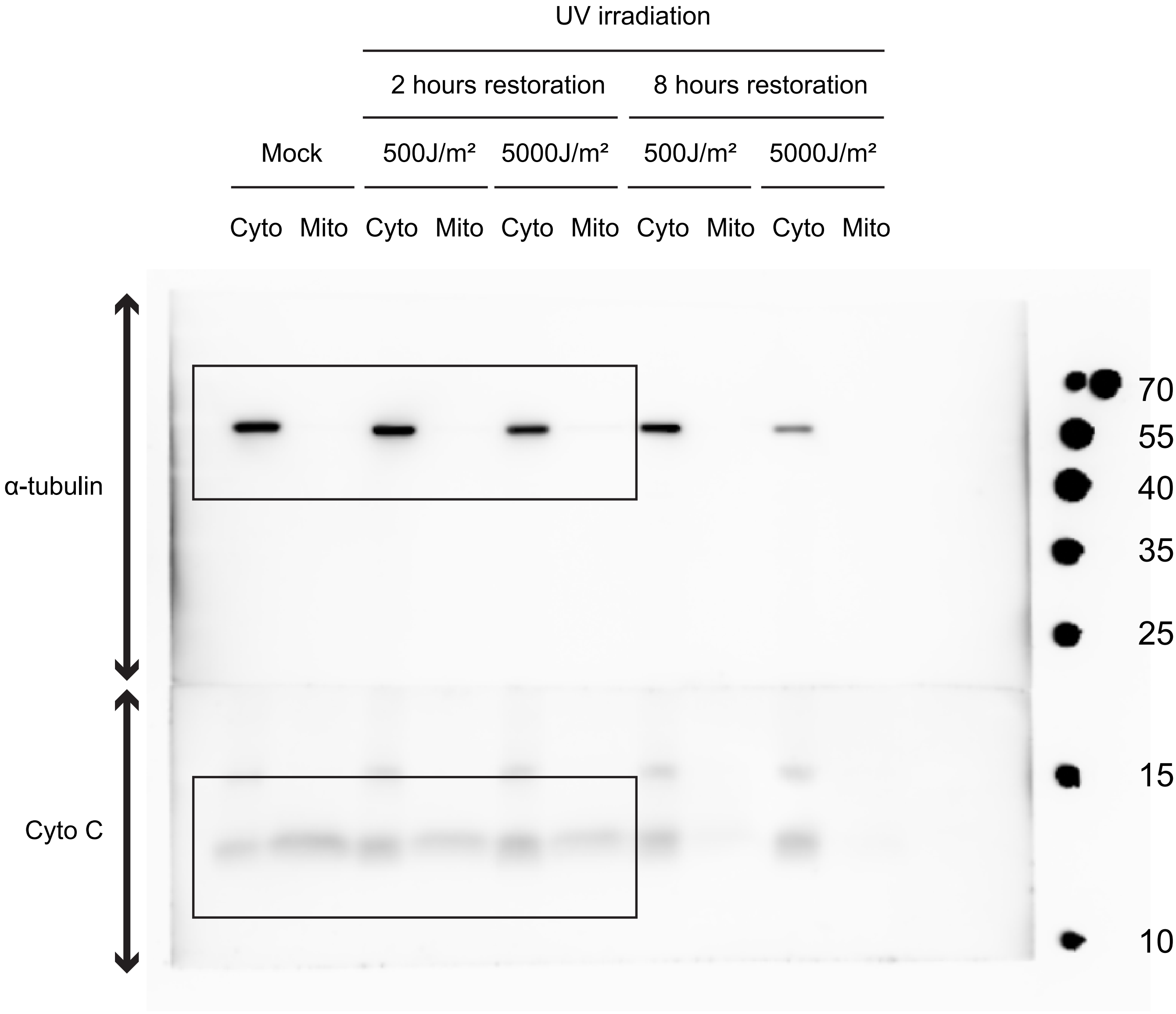

UV irradiation
2 hours restoration
8 hours restoration
Mock
500J/m²
5000J/m²
500J/m²
5000J/m²
Cyto Mito Cyto Mito Cyto Mito Cyto Mito Cyto Mito
α-tubulin
Cyto C
70
55
40
35
25
15
10

Supplementary Figure 17. UV irradiation induces programmed cell death.

(a) Full blot image of membrane from Fig. 6c. Black boxes were cropped and presented in Fig. 6c.

a

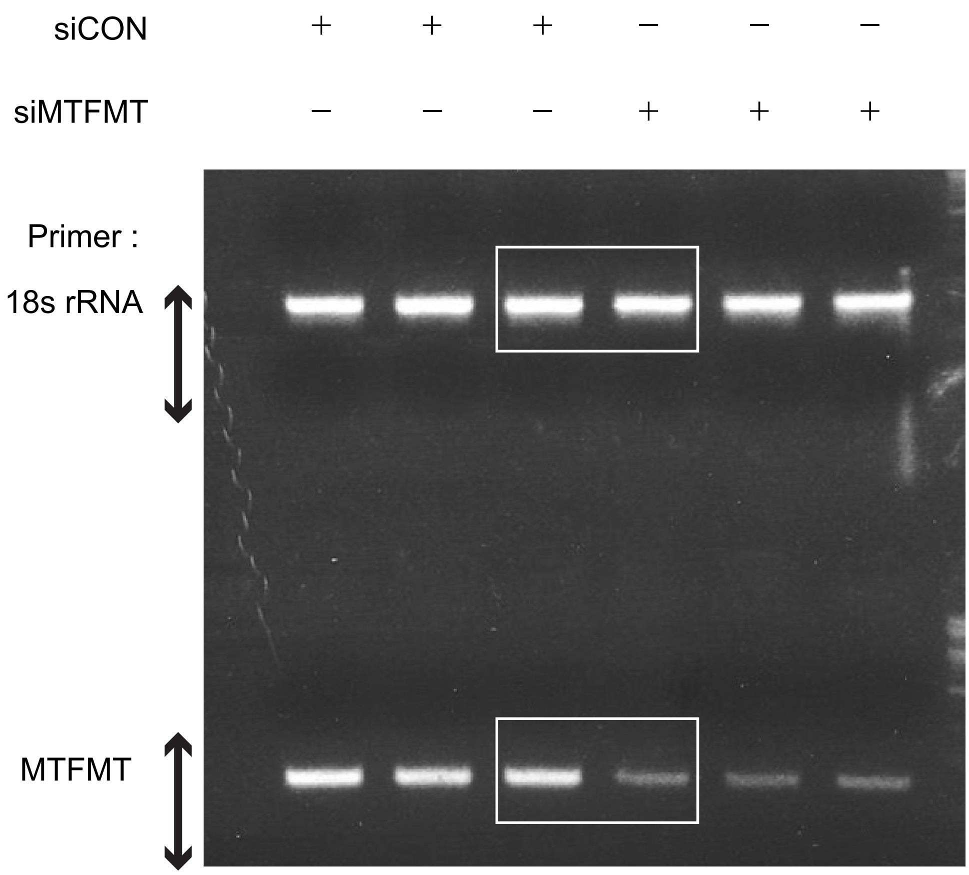

Supplementary Figure 18. Knock-down efficiency of MTFMT is assessed by RT-PCR.

(a) HeLa cells transfected with control or MTFMT were harvested for RT-PCR. The rest of cells were harvested for cell fractionation into cytosolic- and mitochondrial fraction. RT-PCR products were run on 2% agarose gel. After inversion of gel image, white boxes were cropped and presented in Fig. 6d. Samples are listed at the top and primers are listed on the left.

a

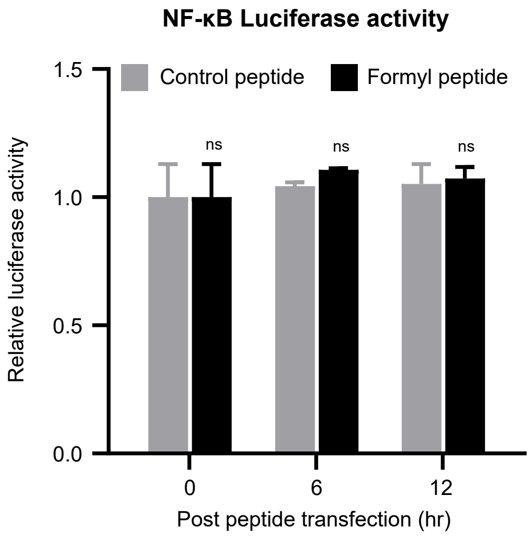
NF-κB Luciferase activity
Control peptide
Formyl peptide
ns
ns
ns
1.5
1.0
0.5
0.0
Relative luciferase activity
0
6
12
Post peptide transfection (hr)

Supplementary Figure 19. Mitochondria-derived *N*-formyl peptide does not directly inhibit NF-κB signaling pathway.

(a) HeLa cells were transfected with NF-κB luciferase reporter plasmid. Forty-eight hours after plasmid transfection, the cells were transfected with a mixture of control or formyl peptides (ND6 + COXI), and luciferase activity was measured at the indicated time points. Error bars indicate SDs and statistical significance was assessed by unpaired t-test.

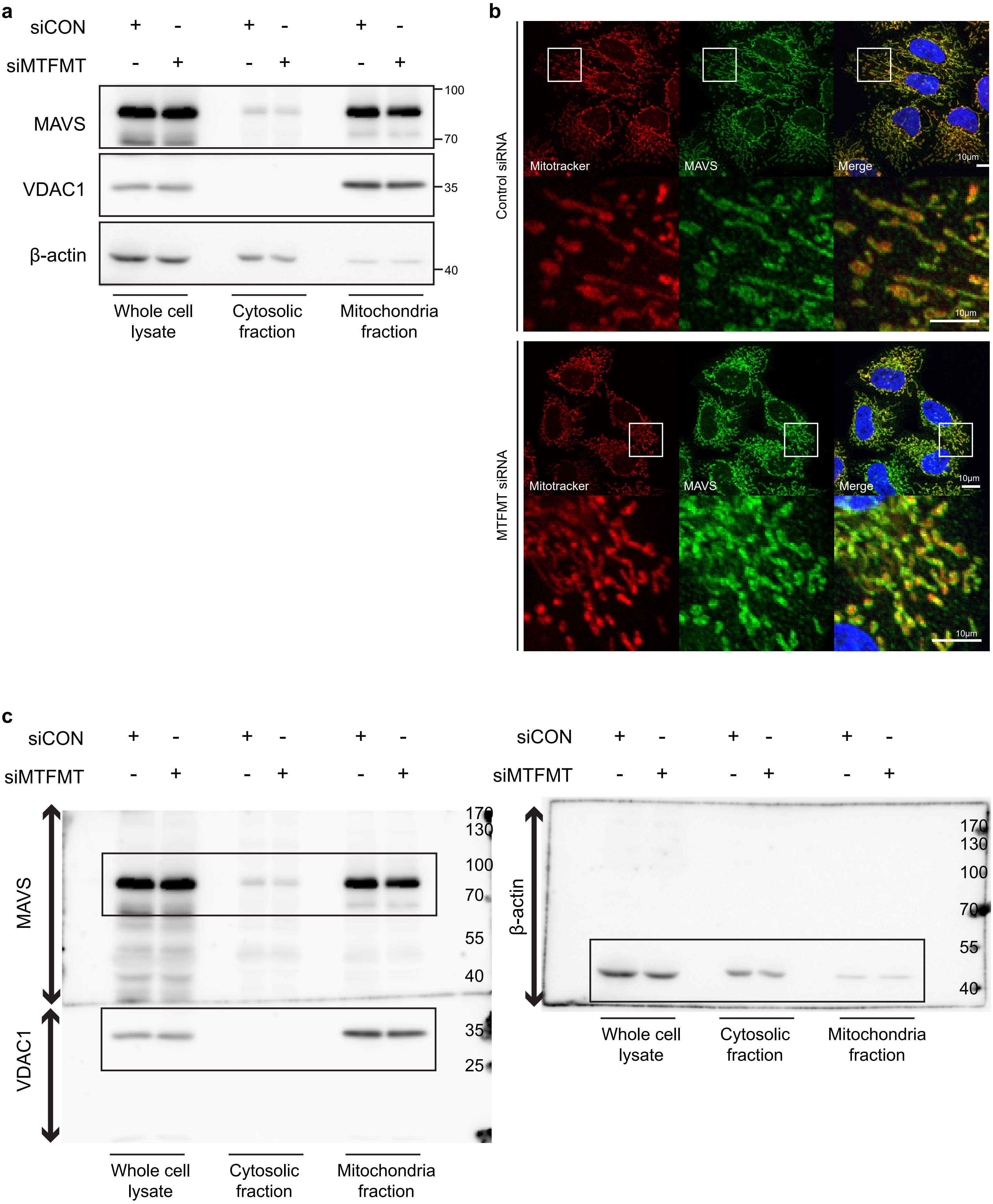
a
siCON + - + - + -
siMTFMT - + - + - +
MAVS
VDAC1
β-actin
100
70
35
40
Whole cell lysate
Cytosolic fraction
Mitochondria fraction
b
Control siRNA
MTFMT siRNA
Mitotracker
MAVS
Merge
10μm
c
siCON + - + - + -
siMTFMT - + - + - +
MAVS
VDAC1
β-actin
170
130
100
70
55
40
35
25
Whole cell lysate
Cytosolic fraction
Mitochondria fraction

Supplementary Figure 20. MTFMT deficiency does not affect mitochondrial accumulation of MAVS.

(a) HeLa cells transfected with control or MTFMT siRNA were fractionated into cytosolic- and mitochondrial fraction as described in Materials and Methods. Samples were run on 9% polyacrylamide gel and membrane was blotted with anti-MAVS antibody to detect endogenous MAVS.

(b) Forty-eight hours after siRNA transfection, HeLa cells were stained with Mitotracker DeepRed for mitochondria and anti-MAVS antibody followed by an incubation with anti-mouse Alexa 488. Nuclei were stained with DAPI.

(c) Full blot image of membrane from a. After stripping of membrane from c, membrane was blotted again with anti-β-actin antibody (right). Black boxes were cropped and presented in a.
